# A multi-stage anticipated surprise model with dynamic expectation for economic decision-making

**DOI:** 10.1038/s41598-023-50529-y

**Published:** 2024-01-05

**Authors:** Ho Ka Chan, Taro Toyoizumi

**Affiliations:** 1 Laboratory for Neural Computation and Adaptation, RIKEN Center for Brain Science, Wako, Japan; 2 Department of Mathematical Informatics, Graduate School of Information Science and Technology, The University of Tokyo, Tokyo, Japan

**Keywords:** Human behaviour, Decision

## Abstract

There are many modeling works that aim to explain people’s behaviors that violate classical economic theories. However, these models often do not take into full account the multi-stage nature of real-life problems and people’s tendency in solving complicated problems sequentially. In this work, we propose a descriptive decision-making model for multi-stage problems with perceived post-decision information. In the model, decisions are chosen based on an entity which we call the ‘anticipated surprise’. The reference point is determined by the expected value of the possible outcomes, which we assume to be dynamically changing during the mental simulation of a sequence of events. We illustrate how our formalism can help us understand prominent economic paradoxes and gambling behaviors that involve multi-stage or sequential planning. We also discuss how neuroscience findings, like prediction error signals and introspective neuronal replay, as well as psychological theories like affective forecasting, are related to the features in our model. This provides hints for future experiments to investigate the role of these entities in decision-making.

## Introduction

The expected utility theory (EUT)^1^ is one of the most well-established models for analyzing decision-making under risk. Nevertheless, throughout the past decades, several experimental works^2,3^ have revealed instances where people’s choice cannot be explained by EUT. Some of those examples with a higher profile are coined the term ‘paradox’. Subsequently, new theories aiming to resolve these paradoxes, e.g. the prospect theory (PT)^3^, the extended cumulative prospect theory (CPT) for actions with multiple outcomes^4^, and the regret theory (RT)^5,6^, have been proposed. Despite the effort, the mechanisms behind decision-making which account for behaviors not conforming to EUT are still an open area of research.

Most of these models focus on dealing with single-stage problems in which no notable events that affect people’s perception of the likelihood of outcomes take place between people making a decision and the revelation of outcomes. However, in real life, decision-making problems often include complex processes such that either the outcome structure, or the people’s perception of such, changes after a decision has been made. In financial investments or trading, the valuation of your investments or trade products is constantly updated amid ongoing events, which affects your potential return. In games like poker and mahjong, you slowly gather information about other players’ hands throughout a game. With the extra information, your assessment of your potential return typically changes, even though you cannot reverse some of your earlier decisions. How post-decision information, or more precisely, the anticipation of receiving such information, affects decision-making is largely ignored in the abovementioned models.

There have been attempts to study isolated problems with more complex structures, e.g. problem 10 in Kahneman and Tversky’s 1979 paper^3^ (to be abbreviated as KT1979). Nevertheless, a general extension of these models to also consider the multi-stage problems outlined in the previous paragraph could be challenging because of some of their typical features. For example, PT and CPT use nonlinear probability weighting functions. However, it is often not easy to decouple the effect of subjective probability perception and evaluation of outcomes^7^. Moreover, studies have shown that the form of mapping between objective and subjective probability, or even the very existence of subjective probability perception, could be highly context dependent^8–10^. The probability weighting function for single-stage problems may change as more events unfold and information is revealed, and this change may be very specific to the nature of the information in each problem. Another feature involves the reference point that people use to gauge the attractiveness of outcomes. While the initial asset position (the baseline level) is conventionally used, recent theories stipulate that the reference point can be at other positions, depending on the contextual nuances of the problem^3^ and the psychological expectation of people^11,12^. As problems become more complex, the determination of a ‘reasonable’ reference point becomes harder and more subjective, and it is unclear whether the use of a single reference point is sufficient.

Some models do employ various restructuring techniques to morph a multi-stage problem into alternative forms, but they revolve around simplifying the problems by merging outcomes, collapsing the problem into a single stage, and thereby eliminating the effects caused by the original problem structure^3^ or adding additional steps for evaluating a particular outcome^13,14^. These efforts do not address the effects of post-decision information on decision-making.

Here, we propose a multi-stage model that explains people’s behavior when facing decision-making problems that involve post-decision information. The basic construction of the model is inspired by important neuroscience findings and psychological theories. First, people may either consciously or unconsciously anticipate possible outcomes of an action before executing it. Many decision-making models explain people’s choices by applying different concepts of ‘anticipation’, e.g. by considering the anticipated regret for not choosing other options^5,6^; by using the accumulation of anticipated elation^15,16^; and by analyzing the possible outcomes a subject anticipate to get^11–13^. Indeed, when animals are planning an action, neurons in the brain exhibit activities that resemble possible task-state sequences^17^. Such sequential neural activities during the anticipation processes was reported to predict the subsequent decisions made^18,19^. In addition, psychological studies on affective forecasting^20,21^ suggest that people also anticipate their future affective states upon occurrence of an event. It is likely that this has implications for decision-making, as hypothesized by some previous work^22^. Second, dopamine neurons in the brain that encode reward prediction errors, i.e., the deviation of the reward outcome from its expected value, are responsible not only for learning but also for motivating actions^23^. Combining these ideas, we hypothesize that the reward prediction error signals encoded during sequential anticipation of task-states is an essential component in decision-making. More specifically, we postulate that, to evaluate each option, people pre-emptively compute along sequential stages how much the expected outcome at the current stage deviates from the expected outcome at the previous stage. This deviation is then nonlinearly scaled by a ‘surprise function’, leading to what we call the ‘anticipated surprise’. The anticipated surprise in each stage is then summed up along the sequences, weighted by the objective probability. The option that maximizes the total anticipated surprise is chosen. In this formalism, the expected value of possible outcomes is effectively the reference point, which dynamically updates along the anticipation stages.

Our sequential anticipation model has some similarity to the one in Kőszegi's 2009 paper^24^. However, there are two key differences. First, their work is a theoretical framework that does not focus on implementation and specifics required for behavioral prediction. While they did construct concrete models as instantiations of their framework, these models do not generate the risk preference explored in our work without including additional factors like probability weighting, which is not straightforward in multi-stage cases. Second, they focus on the effects of ’anticipatory discounting’, which describes the potential diminishing impact of uncertainty in early anticipation stages. By contrast, our model has no discounting parameters of any kind (see "Models with sequential branching and dynamically updating reference points" for a more detailed comparison between the two models).

In this paper, we first introduce in "The skeletal model" our model in its simplest form without sequential anticipation and outline the properties of the surprise function that allows the reproduction of the observations in Problem 3, 4, 7, 8, 14 in KT1979, namely risk-averse for gambling except for lottery type cases in the gain domain, and the reflection effect for the loss domain. In "Sequential anticipation and the full AS model", we show how we build upon the skeletal model by incorporating sequential anticipation to create the full model, which can deal with multi-stage problems. In "Applications of the full AS model", we show how the full model can be applied in different scenarios. First, we look at situations where post-decision information is expected to be explicitly given to the decision-maker. We show that the prediction of our model fits the empirical observations in casino blackjack gambling ("Partial revelation of outcomes: blackjack gambling"). We next argue that the post-decision information need not be actual, but rather, the mental perception of such can also trigger sequential anticipation. It may correspond to some information that people know exists but is not revealed to them ("Ambiguity: Ellsberg paradox (2-urn problem)"), or simply emerge as a by-product of people conceptualizing complicated problems by grouping and segregating events ("Segregation of probable and improbable outcomes"). We show how famous problems like the Ellsberg’s paradox, Allais’ paradox and Birnbaum problem may fall into these categories and be explained by our model. The mathematical details can be found in the Appendices. In "Discussion", we compare our model with other prominent decision-making models and theories, discuss the limitation of our model and suggest possible ways to extend it to consider more general cases of decision-making, and suggest possible neural and psychological mechanisms related to our model.

To avoid confusion among readers, who may have different interests in various aspects of decision-making under risk, we would like to make clear the scope and purpose of the model presented in this work before moving on. First, despite being inspired by neuroscience and psychological findings, the model is primarily a descriptive model that seeks to predict people’s behavior. Second, there have been vast amounts of single-stage problems decision-making models proposed by previous works, which have been documented and classified^25^, and their accuracy been studied^26–28^. Our aim to introduce the single-stage skeletal model in "The skeletal model" is to prepare a building block to create the full model for multi-stage problems, which the models discussed in recent works^26,28^ are not designed to cope with. To better conceptualize the sequential property of this full model, we do not perform detailed parameter fitting or model comparison on the skeletal model using single-stage data. Moreover, to better focus on the role of anticipated surprise, we only consider options with the same or insignificantly different expected values in this work (see "Other systems for outcome evaluation" for discussion on evaluation of options with different expected value). Finally, our work is primarily concerned with decision-making under risk, not decision-making under more general uncertainty. Uncertainty not only arises from risk but also from the ambiguity in probability of outcome occurrence due to various reasons, e.g. incomplete information, limitation in perception and cognition^29,30^ and temporal discounting^16,24^. While in general, our model is not designed to deal with cases in which the probability of outcome occurrence is not well defined, we do have included an example ("Ambiguity: Ellsberg paradox (2-urn problem)") in which ambiguity in probability is modelled by compound lotteries^31^. Please refer to "Aspects of decision-making not considered in this work" for other features of risk preference our model cannot predict (e.g. loss aversion) and other aspects of decision-making not considered in this work.

## The skeletal model

Before going straight into the full anticipated surprise model, which will be abbreviated as the AS model, we will first introduce its skeletal, single-stage form. In the model, ‘surprise’ $z$ is defined by the difference between the “econopsychological” value of an individual monetary outcome in a particular option and the expected value across all outcomes $x$ within that option, i.e. $z= U(x)-{\text{E}}(U(x))$. ${\text{E}}(.)$ denotes the expected value. The “econopsychological” value of money depends on a multitude of factors, like their psychological states, the value of consumption the money brings about, time discounting. These factors are sometimes vaguely embedded in the term ‘utility’. To simplify discussion, we assume that this value is equal to the face value of money throughout this work and drop the function $U$ from now on, i.e. $z= x-{\text{E}}(x)$.

The surprise value $\Delta$ of a choice is then given by

1 $$\Delta ={\text{E}}\left(\delta (z)\right),$$

where

2 $$\delta (z) = \left\{ {\begin{array}{*{20}c} {f\left( z \right)} & {for\,z \ge 0} \\ { - kf\left( {|z|} \right)} & {for\,z < 0} \\ \end{array} } \right.,$$

using an increasing convex function $f\left(x\right)$ defined for $x\ge 0$, and a risk aversion factor $k>1$. At this point, we will just point out the choice of the form of $\delta$ is to allow for famous experimental observations, e.g. the risk-averse behaviors of the decision maker shown when facing 50–50 gamble for even money^32^, and other examples we discuss in later sections. For readers familiar with PT, please note that while the risk aversion factor has a similar counterpart in PT, here $f$ is convex as opposed to the concave value function in PT. In addition, we do not include a probability weighting function as in PT. Please see "Comparison with other decision-making models" for more comparison with PT and "Possible links between anticipated surprise and neuroscience and psychological concepts" for more discussion about the form of the $\delta$ function and its potential link to psychological concepts. Also, as mentioned in the introduction, we only consider options with the same or insignificantly different expected values in this work. With this constraint, options with the highest value of surprise value $\Delta$ are picked.

To get a better understanding of this basic model, let us consider a famous type of gambling problems discussed in KT1979 (Problem 3, 4, 7, 8, 14 and their primed version), as shown in Table 1.

**Table 1:** Outline for the gambling problems in KT1979 (Problem 3, 4, 7, 8, 14 and their primed version). Without loss of generality, we assumed that $p>p^{\prime}$.

	Option 1		Option 2	
	Reward	Probability	Reward	Probability
Outcome 1	$\overline{x }/p$	$p$	$\overline{x }/p^{\prime}$	$p^{\prime}$
Outcome 2	$0$	$1-p$	$0$	$1-p^{\prime}$

In these problems, people have to weigh between an option that gives them a comparatively small amount of money with a large probability (Option 1) and one that gives them a larger amount of money with a lower probability (Option 2). In Table 1, the rewards are scaled such that the expected value $\overline{x }$ is the same for both options. In the actual examples in KT1979, even though this is not always strictly the case, the expected values for both options have always been kept close. We assume that $p>{p}^{\prime}.$

Experimentally, it was found that if the reward is positive (i.e. in the gain domain), most people choose Option 1 (i.e. risk aversion) when $p^{\prime}$ is large and Option 2 (i.e. risk seeking) when $p^{\prime}$ is small. The preference reverses if the reward is negative (i.e. in the loss domain), which is coined the term ‘reflection effect’. It is well known^3^ that this result is inconsistent with the popular EUT^1^ because of the linear relationship between the utility function and $p$.

**Figure 1:**
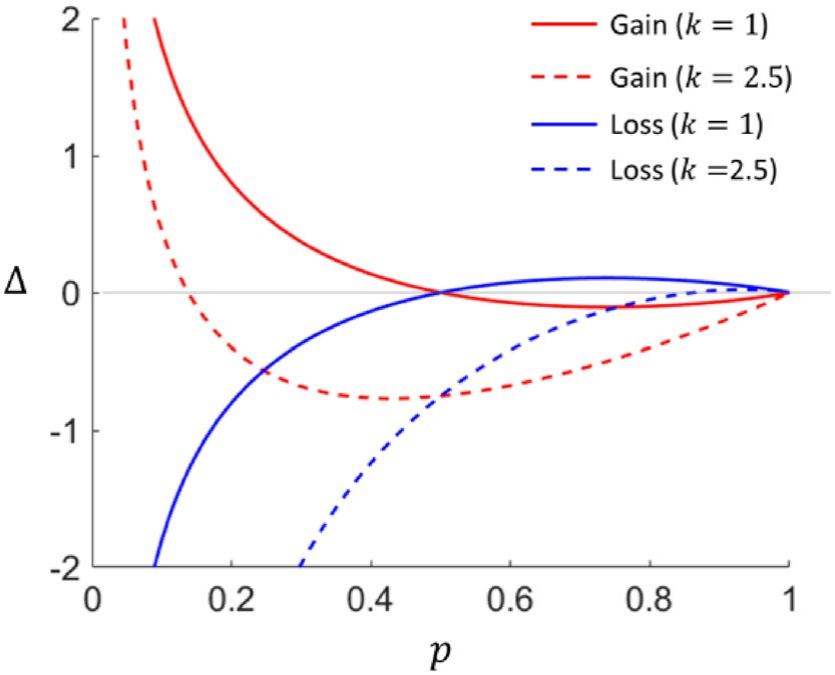
The surprise function $\Delta$ as a function of $p$. In this example, $f(z)={z}^{1.5}$. Red (Blue) lines are the results in the gain (loss) domain. For the solid lines, $k=1$. For the dashed lines, $k=2.5$.

The model can reproduce all response patterns observed in the type of problems described in Table 1 for any convex function $f$. The details of the proof for a general surprise function $\Delta$ is shown in Appendix S1. In Fig. 1, we show how $\Delta$ varies with $p$ using $f\left(z\right)={z}^{1.5}$ as an example. It is trivial that for options that give certain rewards, i.e. $p=1$, there will be no anticipated surprise, i.e. $\Delta =0$. For the gain domain, $\Delta$ decreases from 0 at $p=1$ to negative values as $p$ decreases, indicating that in general people prefer certainty to gambles. However, as $p$ becomes small, $\Delta$ increases and becomes positive, indicating people’s preference for lotteries. When $k=1$, the switch of preference takes place at $p=0.5$, which is much larger than what experimental observations in KT1979 and other works^33^ suggest (Fig. 1, red solid). By picking a larger value of $k$, the switching point can be lowered to a more realistic value of $p$ (Fig. 1, red dashed).

In the model, the reflection effect is observed for all values of $p$ when $k=1$ (see Fig. 1, blue solid, and Appendix S1). When $k>1$, the model can still reproduce the important results of risk aversion (risk seeking) at high $p$ and risk seeking (risk aversion) at low $p$ in the gain domain (in the loss domain, respectively) (Fig. 1). However, the reflection effect becomes imperfect, such that the model predicts risk aversion for both the gain and loss domains when $p$ takes intermediate values (around $p=0.5$ in Fig. 1; see "Other limitations" for discussion on the excessive risk aversion in the loss domain when $k>1$).

## Sequential anticipation and the full AS model

Many decision-making models, including PT, assume a single, static reference point for each problem. Nevertheless, in a problem with sequential structures, people’s expectation generally changes as new information about the outcomes is revealed, which is ignored in PT^34^. This is accounted for in the full AS model. To construct the full model from the skeletal model, we assume that people mentally simulate possible sequences of events comprising intermediate states during decision-making processes and anticipate the change in expectation upon transition to those intermediate states. During the process, the reference point is updated from states to states, based on the expected value of possible outcomes accessible from the new state. The shift of the reference point would also culminate in intermediate surprise.

**Figure 2:**
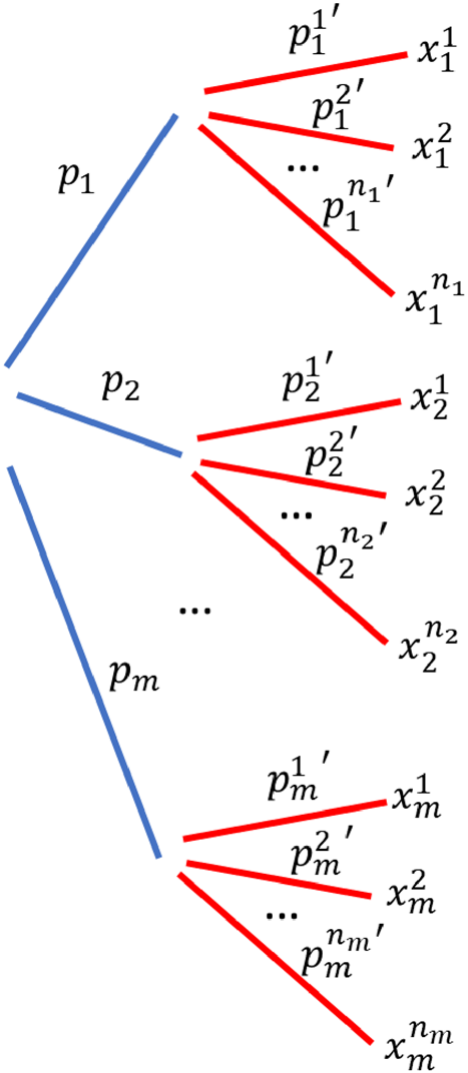
A possible structure of an option in a two-stage decision-making problem.

Figure 2 illustrates how an option in a two-stage decision-making problem may look like.

In this option, there are an intermediate stage and an outcome stage. In the intermediate stage, there are $m$ intermediate states, each happening with a probability ${p}_{i}$, $i=\mathrm{1,2},\dots m$ as depicted by the blue lines. For each intermediate state, it branches out to ${n}_{i}$ possible final outcomes ${x}_{i}^{j},$ $j=\mathrm{1,2},3,\dots ,{n}_{i}$ with probability ${p}_{i}^{j}$ as depicted by the red lines. The surprise value $\Delta$ is given by:

3 $$\Delta =\sum_{i=1}^{m}{p}_{i}\delta \left({E}_{i}-{E}_{0}\right)+\sum_{i=1}^{m}{p}_{i}\sum_{j=1}^{{n}_{i}}{p}_{i}^{j}\delta ({x}_{i}^{j}-{E}_{i}),$$

where ${E}_{0}={\sum }_{i}{p}_{i}{\sum }_{j}{p}_{i}^{j}{x}_{i}^{j}$ is the expected value of all possible outcomes within the entire option and ${E}_{i}={\sum }_{j}{p}_{i}^{j}{x}_{i}^{j}$ is the expected value for the outcomes that is accessible from the ${i}^{{\text{th}}}$ sub-branch (each sub-branch is denoted by a blue line in Fig. 2).

Note that the 1st term in Eq. (3) corresponds to the surprise in the intermediate stage and the 2nd term corresponds to the surprise in the outcome stage. In the model, we assume they contribute additively to the aggregated surprise value.

More generally, $\Delta$ can be computed in a cascading manner. Considering a decision-making process for an option with $T$ stages (including the outcome stage), we define ${y}_{k}(t)$ to be one of the possible state trajectories in our mental branching process, where $t=\mathrm{0,1},\dots ,T$ refers to the hierarchy of the branch and $k=\mathrm{1,2},\dots K$ refers to the index of the trajectory, with $K$ being the total number of trajectories. The surprise value of the $k$th trajectory is given by

4 $${\Delta }_{k}=\sum_{t=1}^{T}p\left({y}_{k}(t)\right)\delta \left({E}_{k}(t)-{E}_{k}(t-1)\right),$$

where $p\left({y}_{k}(t)\right)=\prod_{{t}^{\prime}=1}^{t}p\left({y}_{k}\left(t^{\prime}\right)|{y}_{k}\left({t}^{\prime}-1\right)\right)$ is the probability of observing the $k$th trajectory up to the $t$th stage, with $p\left({y}_{k}\left(t^{\prime}\right)|{y}_{k}\left({t}^{\prime}-1\right)\right)$ referring to the transition probability of entering the state ${y}_{k}\left(t^{\prime}\right)$ given that the previous state is ${y}_{k}\left({t}^{\prime}-1\right)$. ${E}_{k}(t)$ is the expected value of the outcomes that are accessible from the state ${y}_{k}(t)$. Note that ${E}_{k}(T)$ is the actual outcome at the end of trajectory $k$.

The aggregated surprise value is then obtained by linear summation of the surprise value of all trajectories

5 $$\Delta =\sum_{k=1}^{K}{\Delta }_{k}.$$

As in the skeletal model, the option with the largest $\Delta$ is picked. Please note that we are not advocating that the branching process would continue infinitely for all decision problems, because of the obvious cognitive burden it presents. How long would the process go on, whether there would be discount for remote branches, and how the sum in Eq. (5) can potentially be approximated by subsampling trajectories are beyond the scope of this work (see "Computation involved during decision-making" for more discussion).

For some simple problems, the AS model generates the same predictions as models that collapse the problems into single-stage ones. For example, the results of problems 10–12 in KT1979 are also reproduced by the full AS model, because adding a common first stage to the problem of Table 1 does not change the choice according to Eq. (3) (see Appendix S2 for the analysis of our model predictions for problem 10 as an example). However, the model predictions are different with and without the collapsing of sequential branches for general problems.

## Applications of the full AS model

### Partial revelation of outcomes: blackjack gambling

In blackjack gambling, there are two phases of play: the player phase and the dealer phase. At the start of the player phase, the player makes their decision based on the available information on their and the dealer’s hand. At the end of the player phase, the player may receive new information on their hand depending on their earlier decision. While the final outcome may still be uncertain, the probability of the player winning may have changed, which would lead to an update of the expectation of the player. It has been shown that expectation of information about the outcome leads to change in gambling behavior and risk preference^35^, and an update of this expectation also has similar effects^36^. We will illustrate how our full AS model captures the effect of such an intermediate state and leads to correct prediction on some observed gambling behaviors. Please refer to Appendix S3 for the relevant details of the blackjack rules.

Bennis (2004) outlined 3 scenarios in which experienced gamblers face two options with very similar expected return and overwhelmingly choose the slightly worse option. They are 1. standing instead of hitting when the player’s cards totaling 16 points and the dealer’s totaling 10 points (16 vs 10 situations), 2. taking insurance side bets when they have blackjack and 3. taking insurance side bets when they have ‘good hands’, i.e. card compositions that imply a large probability of winning. For simplicity, we make the approximation that the expected returns of both options are exactly equivalent in all the scenarios.

#### Blackjack gambling: 16 vs 10 situations

In this problem, the player may choose to hit (i.e. taking an extra card) or to stand (not taking an extra card). Without any form of restructuring of the problem, the options of hitting and standing are indistinguishable, since for both options, the possible outcomes are equivalent (winning: $$$x$$; losing: $$$-x$$, where $$x>0$$ is the bet size). With the additional constraint that the expected return for both options is the same, it implies that the probability of obtaining those outcomes is also the same. Hence, the preference for standing cannot be predicted by conventional models, including EUT, PT and RT.

**Figure 3:**
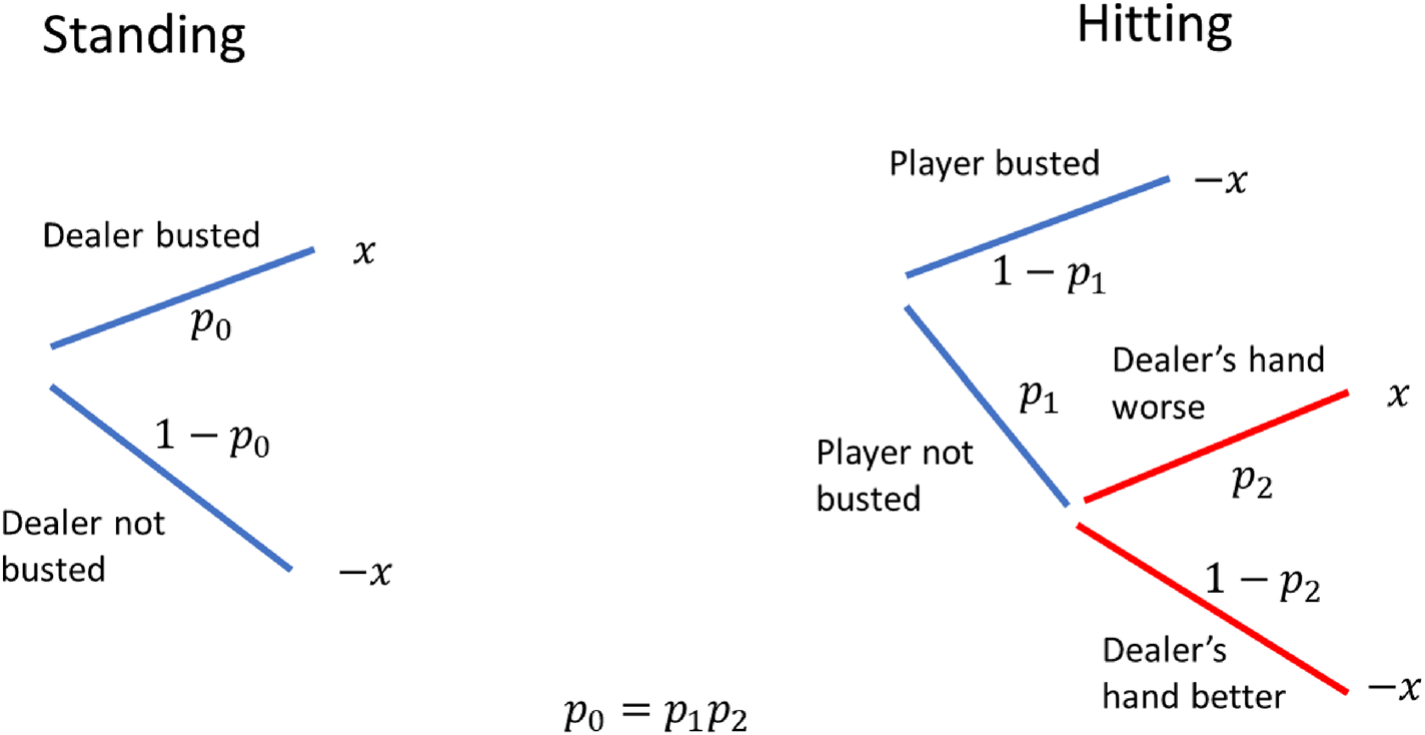
Branching schemes when the player decides to stand (left) and hit (right). Here ${p}_{0}$ is the probability that the dealer goes bust, ${p}_{1}$ is the probability that the player does not go bust after taking a card, ${p}_{2}$ ($0<{p}_{2}<1$) is the probability that the player wins the hand (either because their hand is larger, or the dealer goes bust) provided that they do not go bust. The constraint that the expected value for both options is the same means that ${p}_{0}={p}_{1}{p}_{2}$. We further assume that when the player hits, only one more card will be taken, as taking more cards would lead to significantly worse expected return.

So, how can we understand the observed preference for standing through sequential anticipation? Note that if the player chooses to stand, the player phase is immediately over. The outcome solely depends on what happens in the dealer phase. On the other hand, if the player chooses to hit, the expected returns changes depending on the results of the player phase. If the player busts, the game ends immediately (the player loses). If the player does not bust, the final outcome is still undecided as the dealer has yet to play, but the expected return has now risen because the possibility that the player loses due to busting has been eliminated. The branching schemes for standing and hitting are illustrated in Fig. 3.

The surprise values for standing (${\Delta }_{stand}$) and hitting (${\Delta }_{hit}$) are computed in Appendix S3. It turns out that ${\Delta }_{stand}>{\Delta }_{hit}$ (please see Appendix S3 for the mathematical details.) This result matches the observed behaviors of gamblers.

This example illustrates how branching can influence the preference of people. In the case of hitting, if the player does not bust, their chance of winning increases, meaning that the intermediate state has a higher expected value than the initial state. Thus, the anticipated surprise for winning is split into two small components: one associated with the transition from the initial state to the intermediate state, and the other with the transition from the intermediate state to the outcome state. Because of the convexity of the surprise function, the aggravation of these small components of surprise is less than the big surprise acquired when the player anticipates standing and winning. By similar reasoning, the negative surprise acquired when the player anticipates hitting and losing is more severe than the case of standing and losing.

#### Blackjack gambling: taking side bets when the player has a good hand

In this problem, the player may choose to either take or not take an insurance side bet (i.e. betting that the dealer has blackjack. Please refer to Appendix S3 for details of the rules).

##### Special case: when the player has blackjack

When the player has blackjack, taking the side bet leads to a certain reward of the bet size, while not taking the side bet leads to a reward of 1.5 times the bet size with the probability $\frac{2}{3}$, and 0 otherwise. Note that this is exactly the same decision-making problem as the one depicted in Table 1 with $p=1$, $p^{\prime}=\frac{2}{3}$. We have already established that the certain option, i.e. taking the side bet, is preferred. Sequential anticipation is irrelevant in this case.

##### When the player has a general good hand

**Figure 4:**
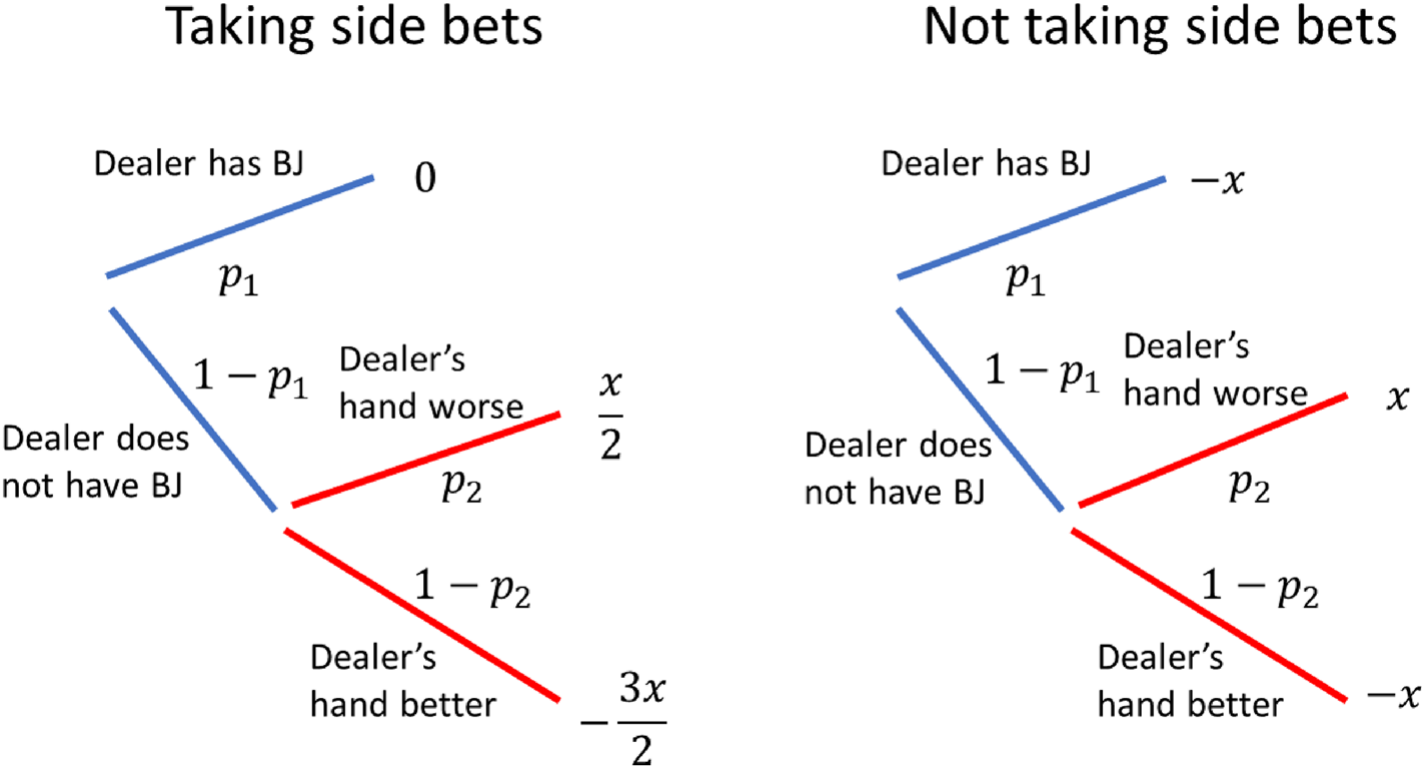
Branching schemes when the player decides to take (left) and not to take (right) the side bet. ${p}_{1}$ is the probability that the dealer has blackjack, ${p}_{2}$ is the probability that the player wins (either because their hand is larger, or the dealer goes bust) provided that the dealer does not have blackjack.

In many casinos, the dealer peeks for blackjack, which means that before the player phase, the dealer checks if their hand is blackjack. If so, the round promptly ends. If not, the expected return for the player increases, since the original expectation has factored in the possibility that the dealer has blackjack and that has been eliminated. This creates a similar branching scheme as in the 16 vs 10 situations with the player hitting, as depicted in Fig. 4.

Since the risk aversion factor $k$ only appears when negative surprise is incurred, the expressions for ${\Delta }_{bet}$ and ${\Delta }_{no bet}$ depend on the value of ${p}_{2}$. We will leave the mathematical details to Appendix S3 and numerical simulation results to Fig. S3 in Appendix S5. Here, we state the results that ${\Delta }_{bet}>{\Delta }_{no bet}$ if ${p}_{2}>\frac{3}{8}$ (note that the converse is not true. The result is inconclusive if ${p}_{2}\le \frac{3}{8}$). Since a large ${p}_{2}$ corresponds to a ‘good hand’, the prediction of the model matches the observed behaviors that people choose to take the side bet when they have a good hand.

To understand the results, first note that the red branches for both options are effectively equivalent, since the deviations from the updated expected values for the outcomes are the same. It makes sense since the side bet is already lost when it is revealed that the dealer does not have blackjack, while the red branch corresponds to the situation after the above-mentioned revelation. For the blue branches, taking the side bet leads to relatively ‘certain’ outcomes. With good hands, the player’s chance of winning is large enough such that it is outside the regime of lottery. In such cases, as we have studied in "The skeletal model", risk aversion dominates, and certain outcomes are preferred.

In Appendix S5, we show that the observed behavior cannot be explained by both PT and RT without considering branching.

#### Final remarks

One may argue that these gambling problems are so complex that the gamblers misinterpret the probability of winning underlying each option. However, information on the ‘optimal strategy’, i.e. the best option to take in order to maximize the expected return, for each initial card composition is well known and publicly available. It has been shown^37^ that experienced gamblers are indeed familiar with such optimal strategies, suggesting that gamblers are likely aware that the options they choose are inferior in terms of expected return.

### Ambiguity: Ellsberg paradox (2-urn problem)

In the above examples, the intermediate states (e.g., if the dealer has blackjack or not) are revealed during the game. Here, we propose that this identifiability of intermediate states is not necessary for sequential anticipation to happen. The intermediate states could remain unidentified and just be a mental product of people conceptualizing a problem. To illustrate this, let us consider the 2-urn version of the Ellsberg paradox^38^. In the experimental set-up, the subjects are told that there are two urns: one containing $$n$$ red balls and $$n$$ black balls; the other containing an undisclosed number of red and black balls totaling $$2n$$. the subjects will then pick a ball in one of the urns. They will win $1 if the ball is red (or black, the color does not matter in the observed behavior) and win nothing otherwise. It has been shown that most subjects prefer to pick balls from the urn with known ball composition. This phenomenon is known as ambiguity aversion.

Let us call picking balls from the urn with known composition ‘Option 1’ and picking balls from the other urn ‘Option 2’. For Option 1, it gives 50% chance of a reward of $1 and 50% of no reward. The surprise value $${\Delta }_{1}$$ is given by:

6a $${\Delta }_{1}=\frac{1}{2}\delta \left(\frac{1}{2}\right)+\frac{1}{2}\delta \left(-\frac{1}{2}\right).$$

For Option 2, it has been proposed that subjects may view it as a compound lottery, i.e. they first envision the various possible compositions in the urn^31^. In the context of our model, these possible urn compositions constitute the intermediate states. The intermediate states can be parameterized by the fraction $$m$$ $$(m=0,\frac{1}{2n}, \frac{2}{2n}, \dots , 1)$$ of balls that are red. Without further information, one may assume that the probability distribution underlying the ball compositions is symmetric about even number of red and black balls, i.e. the probability for entering the intermediate states, $$p$$, is constraint by $$p\left(m\right)=p\left(1-m\right)$$ for all $$0\le m<\frac{1}{2}$$. For each intermediate state $$m$$, there are two final outcomes ($1 with probability $$m$$ or 0 with probability $$1-m$$). Under this formalism, the surprise value $${\Delta }_{2}$$ for Option 2 is given by

6b $${\Delta }_{2}={\sum }_{m}p\left(m\right)\left[\delta \left(m-\frac{1}{2}\right)+m\delta \left(1-m\right)+\left(1-m\right)\delta (-m)\right].$$

**Figure 5:**
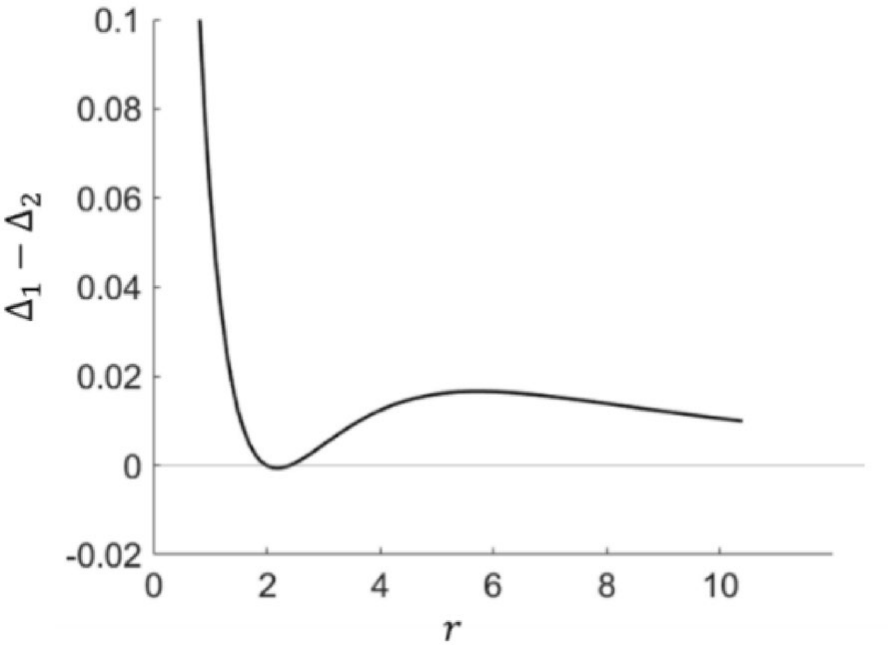
${\Delta }_{1}-{\Delta }_{2}$ vs $r$ for $f\left(m\right)={m}^{r}$ and $p\left(m\right)=\frac{1}{2n+1}$. ${\Delta }_{1}-{\Delta }_{2}>0$ (ambiguity aversion) except for the small regime $2<r<2.5$. $n=50$, $k=2$.

We will leave the mathematical details to Appendix S4. It turns out that ${\Delta }_{1}$ can be larger or smaller than ${\Delta }_{2}$ depending on the choice of $f$, suggesting that both ambiguity seeking and ambiguity aversion is in principle possible. However, we can show that ambiguity aversion is guaranteed if $f$ is not very convex (e.g., $f^{\prime}{\prime}\left(m\right)\le \frac{3}{2}f^{\prime}\left(m\right)$ for $0<m<1/2$) or if $f$ is highly convex (e.g., ${\mu }_{0}f\left(1-{\mu }_{1}\right)>f\left(\frac{1}{2}\right)$, with ${\mu }_{c}=\frac{{\sum }_{m<1/2}{m}^{c+1} p\left(m\right)}{{\sum }_{m<1/2}{m}^{c} p\left(m\right)}$; see Appendix S4). Figure 5 plots ${\Delta }_{1}-{\Delta }_{2}$ using power functions, $f\left(m\right)={m}^{r}$, assuming a uniformly distributed $p\left(m\right)$. Consistent with our analysis, ambiguity aversion is observed both at small and large $r$, corresponding to the regime where $f$ is mildly and strongly convex. In this case, ambiguity aversion dominates over a large parameter space, which echoes the general preference for Option 1 as observed in experiments.

To understand the results, imagine that during our thought process, we first anticipate that the ambiguous urn contains more balls with the prize-winning color than our initial expectation. We would be pleasantly surprised by this potential scenario, culminating in a positive surprise in the intermediate state. Nevertheless, with the expectation now risen, we would then have a negative anticipated surprise going from the intermediate state to the final state because not winning would lead to an outcome much worse than the updated expectation (The 2nd branch is exactly the problems we discussed in "The skeletal model". In this scenario, $p$ is large). In the end, it is a trade-off between the surprise generated in the first and the second transition. The outcome of this trade-off determines one’s affinity for ambiguity.

As we mentioned, we do not have to assume that the subjects are actually informed of the urn composition or its underlying probability distribution for sequential anticipation to take place. How any potential difference in behavior when they are given information of the urn composition, and when they are not, can be theorized, while interesting, is not within the scope of this work (For experimental work on the topic, please refer to e.g. Halevy^39^).

One may argue that when $n$ is large, it is infeasible to mentally consider the huge number of branches as depicted in Fig. 2. In practice, people may evaluate only a limited number of intermediate states such as a single pair of symmetric states, $m$ and $1-m$. As shown in Appendix S4, ambiguity aversion holds robustly in this case (regardless of the form of $p(m)$) if $f$ is not very convex (or if $f$ is highly convex). Similarly, people may use coarse-grained intermediate states to approximate the surprise value.

### Segregation of probable and improbable outcomes

Now we turn to another type of scenarios in which sequential anticipation could possibly occur. Here, intermediate states do not correspond to physical states but reflect mental representations that group multiple improbable outcomes together instead. In real life, there are many events that we may encounter but with very low probability, like earthquake, traffic accident, winning a jackpot. While these events are not ignored altogether, they may not be considered in conjunction with other more probable events^40^. For our model, it means that branches comprising all these rare events are created. To illustrate this, let us consider the original version of Allais paradox^2^ shown in Table 2.

**Table 2:** The two decision-making problems in the Allais paradox.

	Option 1		Option 2*	
	Reward	Probability	Reward	Probability
(a) Problem 1				
Outcome 1	$0$	$0.89$	$0$	$0.9$
Outcome 2	$1$	$0.11$	$5$	$0.1$

	Option 1*		Option 2	
	Reward	Probability	Reward	Probability
(b) Problem 2				
Outcome 1	$1$	$1$	$0$	$0.01$
Outcome 2			$1$	$0.89$
Outcome 3			$5$	$0.1$

The asterisk depicts the option preferred by most people.

**Figure 6:**
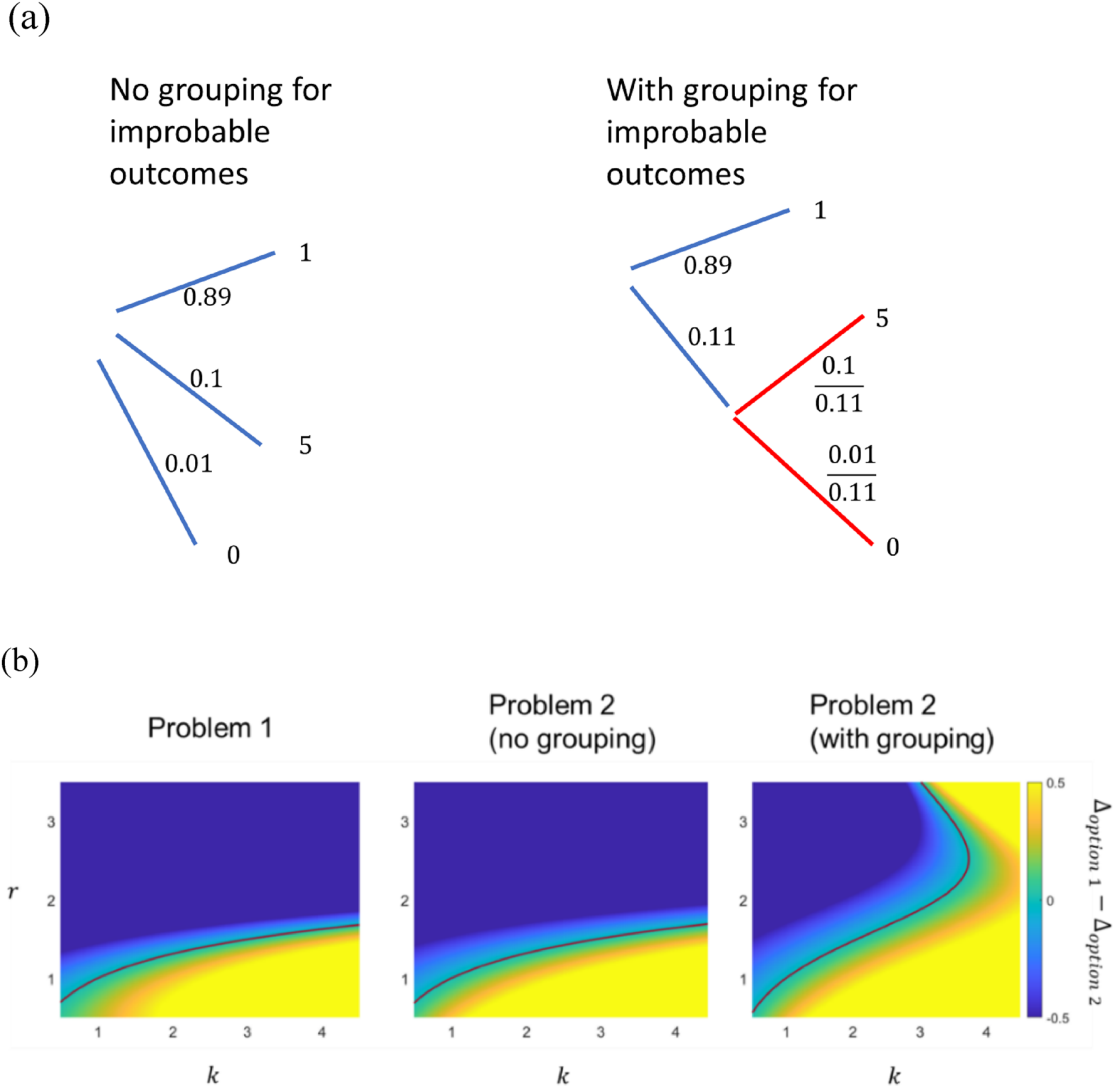
(**a**) The branching schemes for Option 2 of Problem 2 in Table 2 when the improbable outcomes are grouped together and when they are not. (**b**) The difference in surprise values between Option 1 and Option 2 for problem 1 (left), Problem 2 without grouping the improbable outcomes (middle), Problem 2 with the improbable outcomes grouped (right). Yellow (Blue) color corresponds to the regime where Option 1 (2) is preferred. The dark red line is the boundary where the options are equally preferred, i.e. ${\Delta }_{option 1}={\Delta }_{option 2}$. We set $f\left(x\right)={x}^{r}$.

Problem 2 is obtained by replacing the probability 0.89 portion of the reward ‘0’ by reward ‘1’ for both options. Despite this equal treatment on both options, people prefer different options in the two problems. The two options have slightly different expected return, but we assume that its effect is negligible here. First, we will show how sequential anticipation, by splitting the improbable outcomes, ‘0’ and ‘5’ in Problem 2, into sub-branches, makes Option 2 less appealing than the case without splitting. The branching schemes for Option 2 when the improbable outcomes are grouped together and when they are not, are shown in Fig. 6.

The surprise value with (and without) grouping ${\Delta }_{group}$ (and ${\Delta }_{no group}$) are given by:

7a $${\Delta }_{group}=-0.89kf\left({E}_{0}-1\right)+0.1\left(f\left({E}_{1}-{E}_{0}\right)+f\left(5-{E}_{1}\right)\right)+0.01\left(f\left({E}_{1}-{E}_{0}\right)-kf({E}_{1})\right)$$

7b $${\Delta }_{no\, group}=-0.89kf\left({E}_{0}-1\right)+0.1f\left(5-{E}_{0}\right)-0.01kf\left({E}_{0}\right),$$

with the expected values at the initial state ${E}_{0}=1.39$ and intermediate state ${E}_{1}=4.55$. One can easily show that ${\Delta }_{group}<{\Delta }_{no\, group}$ (since $f\left({E}_{1}-{E}_{0}\right)+f\left(5-{E}_{1}\right)<f(5-{E}_{0}$) and $kf\left({E}_{1}\right)>kf\left({E}_{0}\right)+f({E}_{1}-{E}_{0})$ for any convex and increasing $f$ and $k\ge 1$), which means that Option 2 is more unfavorable with grouping than without, implying that the parameter space where Option 2 is worse than Option 1 becomes larger when grouping is considered.

Next, using a common class of convex functions $f\left(x\right)={x}^{r}$, we compare the surprise values of the options for both problems numerically (Fig. 6b). For Problem 1, Option 2 is preferred for most regimes (only except when $f$ is weakly convex and $k$ is large). For Problem 2, the preference remains unchanged in contrary to the experimental observation when grouping is not considered. However, when grouping is considered, in line with our analysis, the surprise value for Option 2 reduces (see Fig. S4 in Appendix 6) such that Option 1 is now preferred for a sizable parameter space. Our model is consistent with the experimental observation either when $k$ is not too large ($k<3$) and $f$ is moderately convex ($1<r<2$), or when $k$ is very large and $f$ is very convex.

Please note that the observations in Table 2 is not considered inconsistent by CPT and RT (see Appendix S4). In fact, Problem 3, 4, 7, 8 in KT1979 are sometimes considered as variants of Allais paradox. However, our model explains them using different mechanisms: the problems in KT1979 by the convexity of the surprise function and the original version of Allais paradox by grouping of improbable outcomes.

Outcome grouping is relevant for a wide range of decision-making problems beyond variants of Allais paradox, for instance when there are multiple events that give the same outcome. In EUT and CPT, such events are effectively combined into a single event. In other words, these models predict that experimentally, combining same-outcome events makes no difference in the observed behavior. However, this is not necessarily true, as illustrated by an experiment in Table 1 of Birnbaum’s 2008 paper^41^ shown in Table 3.

**Table 3:** The two decision-making problems in Table 1 of Birnbaum’s 2008 paper.

Option 1			Option 2*		
Color of ball drawn	Reward	Probability	Color of ball drawn	Reward	Probability
(a) Problem 1					
Red	$100$	$0.85$	Black	$100$	$0.85$
White	$50$	$0.1$	Yellow	$100$	$0.1$
Blue	$50$	$0.05$	Purple	$7$	$0.05$

Option 1*			Option 2		
Color of ball drawn	Reward	Probability	Color of ball drawn	Reward	Probability
(b) Problem 2					
Black	$100$	$0.85$	Red	$100$	$0.95$
Yellow	$50$	$0.15$	White	$7$	$0.05$

The asterisk depicts the option preferred by most people.

**Figure 7:**
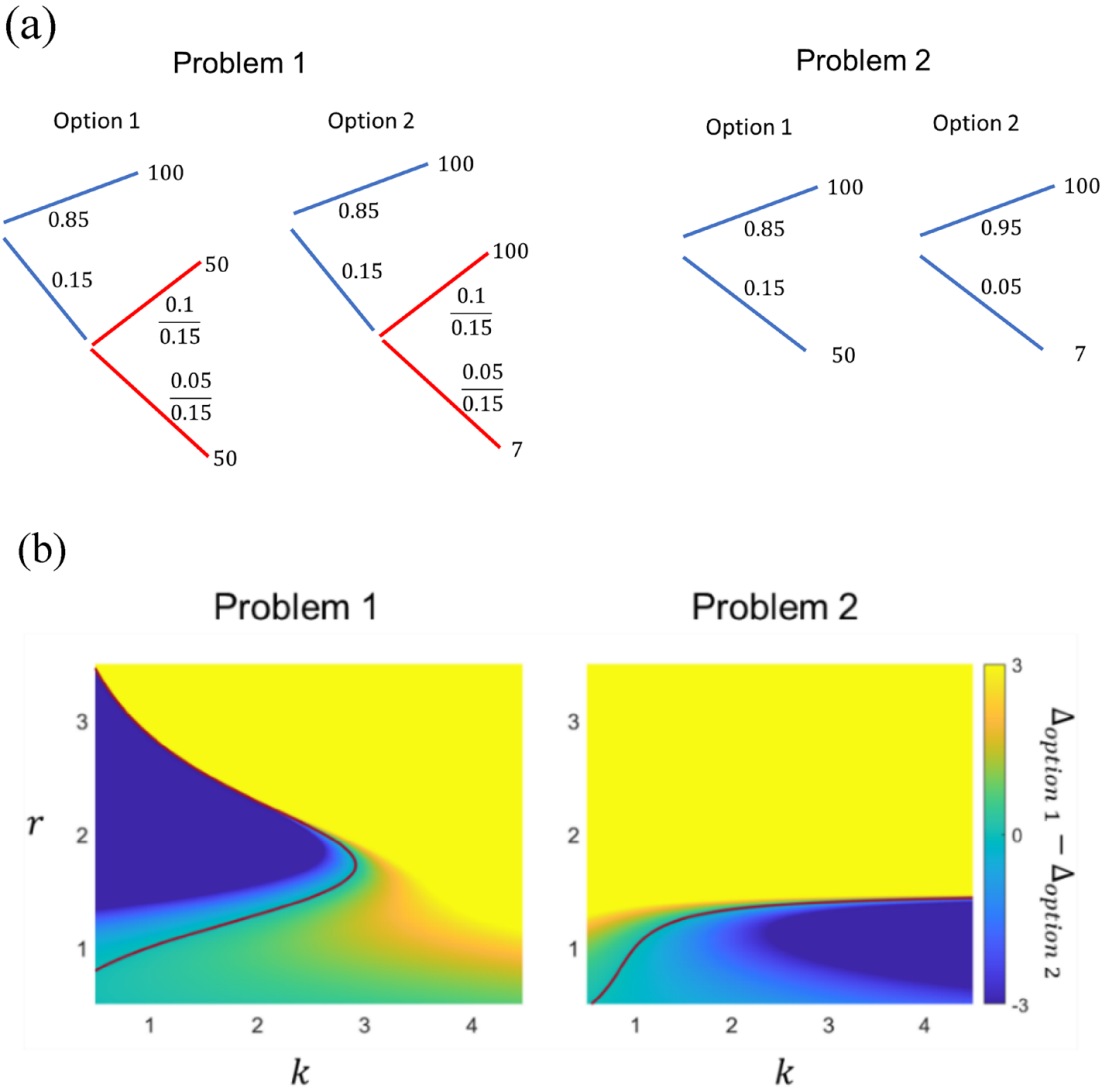
(**a**) The branching scheme for Problem 1 (top) and Problem 2 (bottom) in Table 3. (**b**) The difference in surprise values between Option 1 and Option 2 for Problem 1 (left), Problem 2 (right). As in Fig. 6b, yellow (Blue) color corresponds to the regime where Option 1 (2) is preferred. The dark red line is the boundary where the options are equally preferred, i.e. ${\Delta }_{option \,1}={\Delta }_{option \,2}$. we set $f\left(x\right)={x}^{r}$.

Although the two problems are identical if the same-outcome events are combined, most people choose different options for Problem 1 and Problem 2. This motivates us to again investigate the branching schemes that group the improbable events and without combining the same-outcome events, as shown in Fig. 7a. Again, we neglect the effects of different expected returns for the two options (c.f. “Discussion”).

For Option 1 in Problem 1, the events that lead to the same outcome are both improbable, and therefore get grouped into the same branch. In this case, they can be combined since the branch they are in leads to a certain outcome, i.e. no ‘surprise’. In other words, Option 1 of Problem 1 and 2 are equivalent. For Option 2 in Problem 1, one event with the reward of ‘100’ is probable while the other is improbable, causing them to be grouped into separate branches. In this case, the branches cannot be combined (note the similarity to Fig. 6a).

The surprise values for Option 2 in Problem 1 (${\Delta }_{group}$) and Problem 2 (${\Delta }_{no\, group}$) are given by

8a $${\Delta }_{group}=0.85f\left(100-{E}_{0}\right)-0.15kf\left({E}_{0}-{E}_{1}\right)+0.1f\left(100-{E}_{1}\right)-0.05kf({E}_{1}-7)$$

8b $${\Delta }_{no \,group}=0.95f\left(100-{E}_{0}\right)-0.05kf\left({E}_{0}-7\right),$$

with the expected values at the initial state ${E}_{0}=95.35$ and intermediate state ${E}_{1}=69$. To explain the experimental results, ${\Delta }_{group}>{\Delta }_{no \,group}$ should hold. This is true if $k=1$ due to the convexity of the surprise function $f$ in the gain domain, similar to the case in the Allais paradox. More generally, when $k>1$, ${\Delta }_{group}>{\Delta }_{no\, group}$ requires that the effects of $f$’s convexity overwhelm that of the risk aversion factor $k$. We illustrate this by comparing the surprise values of the two options for both problems numerically. As the consequence of event grouping, the surprise value for Option 2 increases as long as $r$ is not too small (See Supplementary Fig. S4 in Appendix 6), such that the model can reproduce the experimentally observed preference when $k$ is not too large ($k<3$) and $f$ is moderately convex ($1<r<3$) (Fig. 7b). Note that this regime roughly overlaps with the regime where our model’s prediction is consistent with experimental observation for the Allais paradox we previously discussed (at roughly $2<k<3$ and $1.2<r<1.8$).

In both the Allais paradox and the Birnbaum problems, by grouping improbable events together in a main branch and moving the detailed anticipation of individual events to separate sub-branches, the associated surprise is broken down into a general one about the group and a specific one about individual events within the group. Since the surprise function is nonlinear, it leads to changes in the aggregated surprise, and hence the preferred options.

## Discussion

### Summary of the work

In this work, we introduced a multi-stage decision-making model that takes the mental perception of post-decision information into consideration. We first created a skeletal model of anticipated surprise. This skeletal model hinges on 3 main assumptions: 1. The reference point being the expected value of outcomes; 2. The surprise function is convex in the gain domain (and concave in the loss domain) and 3. general bias towards risk aversion ($k>1$). It can explain Problem 3, 4, 7, 8, 14 in KT1979.

We then extended the skeletal model into the full model by incorporating sequential branching structures arisen from people’s mental anticipation of possible sequences of events and considering the update of reference point from one stage of another. We show that this sequential anticipation is applicable not only when the post-decision information is explicit, but also when they are expected to be not revealed and when they are just an abstract mental product that emerges when we evaluate probable and improbable information in separate groups The full model can predict behaviors in problems that are considered equivalent by single-stage models. Our numerical results and mathematical analysis show that in order to replicate the experiments, the surprise function needs to be moderately convex, and $k$ needs to be larger than 1 but not too large. This is generally in line with the assumption of the model we made when we considered the single-stage problems in "The skeletal model".

### Comparison with other decision-making models

#### Prospect theory and related works

One of the most striking differences between PT and the AS model involves the mechanism for reproducing people’s risk preference observed in Problem 3, 4, 7, 8, 14 in KT1979. The fundamental reason why EUT cannot reproduce the observed risk preference in these problems is that using a baseline reference point, the convexity (or concavity) of the utility function alone can only lead to either risk-seeking or risk-averse behavior irrespective of $p$. PT and CPT tackle this problem by introducing probability weighting to counteract the effect of nonlinearity in the utility function. More specifically, the overweighting of small probability is essential for reproducing the risk preference in this regime. The logic of this solution also applies for models^42^ that implicitly introduce effective probability weighting. By contrast, through using the expected value of the outcomes as the reference point, our model can reproduce the risk preference at both large and small $p$ by mere convexity (concavity) of $\delta$ in the gain (loss) domain without resorting to probability weighting. One issue with probability weighting is that, as discussed in Introduction, subjective probability perception is highly context-dependent^8–10^, meaning that any probability weighting function may only be applicable to a very limited set of problems.

PT introduced several editing rules to account for some anomalous behavioral observations. While there are guidelines on how to apply them, a large degree of freedom for the usage of the rules remains. There are situations, e.g. in the original version of Allais paradox^2^, in which multiple editing rules are eligible. The choice of which rules to apply and the order of their application could lead to predictions of different behaviors^41^. Moreover, the only way PT and CPT deal with multi-stage problems is by collapsing the stages using those editing rules, which is not possible in most cases. While there could be other ways to reduce these problems to single-stage ones, they often involve computation of complex statistics and weighting functions^43^. On the other hand, sequential anticipation in our model not only resolves some ambiguities in the editing rules (see e.g. "Segregation of probable and improbable outcomes"), but also provides a well-defined mechanism that allows general multi-stage problems to be studied in their original form. One may ask if similar sequential branching mechanisms could be introduced in PT and CPT. However, it is non-trivial to formulate the Markovian transitions of the intermediate states under their framework due to their use of nonlinear probability weighting.

#### Models using endogenous reference points based on psychological expectation

Several recent models use ‘expectations’ in a more general sense as reference points. These expectations can either be determined by subjective analysis of the problem contexts^11–13^ or by objective measures, e.g. one or more outcomes in a problem^5,6^ or statistics derived from them^44^. While we believe that the psychological processes that underlie these reference points do affect people’s choice, as demonstrated by the works cited above and empirical evidence^45^, other laboratory^46^ and field^47^ studies have shown the mathematically-based expected value is also highly relevant in decision-making. Intuitively, when people are put into situations that lack contexts and that they have no prior experience with, there would be little reason for them to have specific goals or aspirations, leaving the objective expected value the only logical type of ‘expectations’ they can have.

From the neuroscience perspective, the expected value plays a key role in gauging rewards. An evidence is that the activities of dopamine neurons, which are well described by reward prediction errors^48–51^, are relevant to behaviors in the presence of potential reward^23^ (more discussions in "Possible links between anticipated surprise and neuroscience and psychological concepts"). Thus, using the ‘objective’ expected value as the reference point has the advantage of adding clear and well-founded biological relevance to our model.

#### Models with sequential branching and dynamically updating reference points

To our knowledge, the only work that models problems using a sequential branching structure with dynamically updating reference point is Kőszegi’s 2009 paper^24^. Their work outlines a generic framework that primarily investigates the role of anticipatory discounting of early stages. It does not specifically discuss the implementation of the model (e.g. what possible form the utility function may take, whether there would be subjective probability). One possible instantiation of the model based on some examples in their previous work^11,12^ involves making a rank-based comparison over whole range of possible outcomes people anticipated before and after information-providing events, which is then scaled by a concave utility function. It cannot reproduce some of the observed risk-seeking and risk-aversive preferences (e.g. Problem 14 in KT1979 and its reflection effect) unless probability weighting is also introduced. (As we mentioned in "Prospect theory and related works", probability weighting becomes non-trivial when sequential branching is considered). Another important feature of this instantiation is that adding new non-trivial stages always results in a decrease in utility. An increase in utility can only be achieved by introducing additional ’anticipatory discounting’ parameters that mitigate the impact of uncertainty in early anticipation stages. However, determining these anticipatory discounting parameters could be challenging, since mental anticipation of events may not follow their chronological order^52^. It may also just reflect how we mentally structure a problem and its outcome^40,53^, and thus be unrelated to the timing of information revelation (see the examples in Birnbaum^41^ and the discussions in Philips et al.^40^). In contrast, our model can both predict a decrease (e.g. blackjack 16 vs 10) and an increase (e.g. the Birnbaum problems in section "Segregation of probable and improbable outcomes") in utility when adding new stages and sequential branches without introducing such parameters. Regardless of the potential relevance of anticipatory discounting in outcome evaluations^15,16^, the simple construct of our model, i.e., without probability weighting and any kind of discounting parameters, might be adequate to offer important insights about the role of sequential anticipation in decision-making.

### Aspects of decision-making not considered in this work

#### Other systems for outcome evaluation

Our work proposed ‘anticipated surprise’ as one of the (potentially many) entities that influences outcome evaluation, and hence decision-making. We do not preclude the co-existence of other brain systems and mechanisms that are also responsible for evaluating outcomes. The fact that the AS model ignores the differences in expected value between options already hints at an alternative system that focus on the said expected value difference. It is known that people are capable of making decisions that are heavily based on expected value when trying to suppress their emotions, e.g. professional poker players playing poker^54^. It is therefore reasonable to speculate that the abovementioned alternative system is more rational and analytic in nature, contrasting to the AS model, which could be more emotionally based (see "Possible links between anticipated surprise and neuroscience and psychological concepts" below for the potential link of our model to affective forecasting). How these different systems interact with each other to form the final evaluation is an interesting research direction to pursue.

#### Computation involved during decision-making

Our work does not investigate the implementation details of the evaluation process of an option, i.e. how the ‘anticipated surprise’ is computed in the brain. Some previous works focus on the computation aspects of decision-making that involve anticipation and forward simulation. For example, decision field theory^14,55^ proposes that when facing uncertain options, we mentally simulate many instances of the potential course of events, each with slight variations and sometimes simplifications in the outcome made. All these instances contribute to the total utility, and decisions are made, for instance, using a threshold system. There are no apparent reasons why a similar process cannot be applied to the computation of the ‘anticipated surprise’, so our model and the central ideas of decision field theory are not mutually exclusive. That said, the examples in this work can be explained by the AS model without having to add variations to the outcomes.

One concern about the mental simulation involved in the AS model is that real-life problems are often very complicated, corresponding to a highly elaborate branching structure that is impractical to compute. How the brain creates representations of the world that are sufficiently accurate and flexible, while at the same time computable, is a topic frequently researched. For example, reinforcement learning studies^56,57^ show that depending on contextual details, people are capable of using both the efficient but less flexible model-free reinforcement learning and the flexible but computation-heavy model-based reinforcement learning when making sense of the structure of a problem and evaluate the different states within it. There are also theories, e.g. successor representation^58^, that proposes a modified form of model-based reinforcement learning that simplifies the structures of a problem by only retaining its essential features. These works suggest that people have a repertoire of strategies for conceptualizing the futures states they may find themselves in and may adopt one that tailors to the decision-making problems at hand.

Another concern is how the computation involved in the AS model could be efficiently performed in neural systems. For complex problems, even after using the simplification processes described in the previous paragraph, the branching structure may still necessarily be extensive. It may seem burdensome and time consuming to go through every branch and compute both the expected value and anticipated surprise for each node and sub-branch. We hypothesized this can be partially resolved by parallel computing, especially for experts with appropriate training. A recent work^59^ presented findings that are suggestive of parallel computing by dopamine neurons. The diverse response profile of dopamine neurons allows them to simultaneously encode the surprise associated with multiple probabilistic outcomes. On the other hand, parallel computing implemented in brain areas relevant for planning and action selection, like the basal ganglia^60^, may enhance the efficiency of searches through branching trees^61^, which has practical importance in decision-making situations in which decisions have to be made under time pressure^62^.

#### Individual variabilities in decision-making

In the examples presented in this work, we focus on decision-making on the population level. In reality, decisions under risk and uncertainty are seldom unanimous, even for the famous example of a 50–50 gamble for even money^3^. One plausible explanation is that the parameters $$k$$, $$r$$ of some individuals take such outlying values that the model behavior changes qualitatively (e.g. $$k<1$$, $$r<1)$$. Regarding the typical range of the parameters, while this is beyond the scope of this work, one may speculate that, given the predictions of the model on the Allais’ paradox and Birnbaum problems described in "Segregation of probable and improbable outcomes", for the majority of people, $$k$$ and $$r$$ would take moderate values larger than 1, e.g. at roughly $$2<k<3$$ and $$1.2<r<1.8$$.

One may also ask whether the parameters of one subject are correlated across different decision-making contexts. This is again beyond the scope of this work, but as a speculation, we believe they will be somewhat correlated, given that similar parametrical studies^63^ in PT suggest that parameters are dependent on invariable qualities of a person, e.g. their cultural upbringing, gender. However, this correlation would not be very strong, as contextual factors, e.g. emotional states^54,64^, social environements^54^ and past decisions^65^ have been shown to have a significant bearing on decision-making. A more definitive answer will require dedicated experimental work on the respective topics, which we will leave for the future.

#### Other limitations

A possible weakness of our model is that it may predict excessive risk-averse behavior in the loss domain. For example, it leads to the prediction that people would take the more certain options for both the gain and loss regime at around $p=0.5$ (Fig. 1) when $k>1$, which could be at odds with some experimental findings^3^. Nevertheless, generally speaking, preferences of people tend to be ambiguous at intermediate values of $p$^33,63^. Even at medium-large value of $p$, risk-averse behavior could still be observed in the loss domain under some set-ups^66^. So, qualitatively speaking, the partial lack of reflection effect predicted by our model in this regime may not be a fundamental weakness of the model. Another related problem is the ‘Asian disease problem’^67^, in which people essentially change from being risk-averse to being risk-seeking when the description of options emphasizes the loss instead of the gain in the possible outcomes. A common way of formulating the problem is by shifting all outcomes by a constant value as in Tversky and Kahneman^67^. Our model in its current form cannot reproduce the correct risk choice because anticipated surprise is computed only in relative to the expected outcome, which also shifts along with the individual outcomes. However, there are multiple possible ways to modify our model such that it could explain the risk-seeking behaviors in the loss domain. For example, one could make $k$ (the risk aversion factor) a variable which is decreasing with the expected outcome, so that as the expected outcome becomes negative, $k$ becomes small, and hence the decision maker becomes more risk-seeking. Alternatively, the reference point might be slightly reduced from the expected outcome in the loss domain to alleviate the disappointment when getting a bad outcome and boost the surprise when getting a good outcome in a gamble. In a word, these problems alone do not refute the basic hypothesis of our model that expected outcome determines the reference point for economic decision-making problems.

### Possible links between anticipated surprise and neuroscience and psychological concepts

Our model is based on two concepts: ‘surprise’ and ‘anticipation’ about reward outcomes. Both concepts have strong neural and psychological bases. In neuroscience, reward prediction error is closely linked to dopamine neurons. The activities of dopamine neurons encode the deviation of the actual reward from the expected reward^48,49,51^, which can be well approximated by the expected value across all possible rewards^50^. As for anticipation, it has been shown that shortly before an animal executes an action, hippocampus place cells exhibit a sequence of firing patterns that is highly predictive of the subsequent action sequence^17,19^. This suggests that these anticipative patterns may be relevant to planning and deciding what actions to take by the subject. Such anticipation-related neural activities are observed in multiple brain areas, including the hippocampus, the neocortex, and the dopaminergic midbrain. Integrated information from these areas is predictive of the decisions made by people^16^.

In psychology, there are theories suggesting that limitation in people’s perception^29^ and cognitive abilities^30^ could explain many features in PT, e.g. its concave value function and S-shaped probability weighting function, as well as some other observed risk preferences in an intuitive manner. The multi-stage problems our model tackles are largely outside the scope of PT, and applying these existing theories to understand such problems is not straightforward. While this may be possible (for instance, one may hypothesize that structurally more complex problems should increase uncertainty in perception and cognition, which may lead to more aversive behavior. This remains to be tested), it is also useful to consider alternative psychological mechanisms that can more easily relate to the features of our model, surprise and anticipation. ‘Surprise’ is often considered to be an emotional state. The notion of people anticipating their future emotional states caused by the outcomes in events is closely related to affective forecasting^20,21^. It is widely thought that affective forecasting has an impact on decision-making, including for the case when the outcomes are uncertain^22^. Interestingly, such ‘emotional anticipation’ often exhibits biases. For instance, it has been shown that people tend to overestimate the intensity and duration of their emotional response^20,68^, in particular for the loss regime^22,69^. These biases may offer an explanation for the inclusion of the loss aversion factor $k>1$ and the convexity of $\delta$ in the surprise function. The hypothesis made in our model and its potential relations with affective forecasting provides a new perspective for interested psychologists to look into.

As a final note, decision-making may involve several mechanisms with different underlying psychological processes, which may be reflected in the various types and forms of ‘utility functions’ proposed in different models and theories. For example, the ‘utility’ in Ref.^29^ concerns people’s internal transformation of the amount of money from the actual amount to a perceived amount, while the ‘utility’ of our model (i.e. $\Delta$) concerns people’s anticipation of their emotional valence derived from the disparity between the expected value of the possible amount of money they could have received and the actual amount received. It is possible that they are referring to different aspects in decision-making and both contribute to shaping the rich behavioral patterns observed in single-stage and multi-stage problems. The potential relationship behind these different utility functions and their roles within the decision-making process should be clarified by future studies.

### Effect of learning on evaluation of anticipated surprise

The problems we studied are hypothetical in the sense that the probability and the size of rewards are explicitly given (Blackjack could be considered an exception, though the probability of winning for each card combination and for each option to take is widely available). In real life, these elements may have to be learnt through trial-and-error. There is ample evidence that learning and past experiences affect decision-making. It has been shown^70^ that offline replay, corresponding to learning and memory consolidation, alters the subsequent decision made during tasks. In practice, the type of decisions made in the past provide a ‘context’ that systematically alter the risk preference for subsequent decisions^65^. For instance, a trader’s return in the recent past affects their risk attitude and expectation on future return, even though it is often unclear whether past return is indicative of future return due to the volatility of the financial market^71^.

How this learning is implemented in conjunction with decision-making under risk is outside of the scope of the model in this work. Simplistically, one could use one of Bayesian reinforcement learning frameworks to train an “anticipated surprise model” for risky decision-making. Nevertheless, it is unlikely that learning and decision-making can be completely decoupled. Based on the similarity of online replay (during task performance) and offline replay (outside task performance), it is strongly suggested that that similar neural activity patterns are involved in planning and learning^17^. Some previous works^16,72^ have proposed model-based reinforcement learning models in which the anticipated prediction error, or ‘surprise’, contributes to the reward values and influences learning. Moreover, the magnitude of anticipated surprise likely changes along the learning stages. For example, it has been shown^73^ that both the happiness for winning a small price and sadness for losing in a lottery is smaller for people who have bought a ticket than those who have not bought a ticket but rather given one. Since inevitably, there were people who regularly buy tickets, and therefore frequently experienced winning and losing, in the group of ticket buyers, the results could possibly be interpreted as desensitization of emotions by repeated exposure to certain events. Indeed, it has been shown that responses from dopamine neurons, which modulate emotional processing, habituate with repeated presentations of the same stimuli^74,75^. Given the role of dopamine neurons in relation to decision-making and encoding reward prediction errors, it is not unreasonable to speculate that such repeated stimulation may have a similar desensitizing effect on anticipated surprise. How the model presented in this work can be extended to include the effect of learning decision-making could be an interesting future topic to study.

### Future experiments to validate the full AS model

As we have shown, our model and its sequential anticipation mechanism allows us to have new understandings on the problems in "Applications of the full AS model". It would be useful to think about what kind of new experimental data one should collect in order to further validate the model on more general problems. In our model, decisions in multi-stage problems often predominantly depend on just a few branches because of the convexity of the surprise function. This constitutes an experimentally testable prediction, since given a problem with a particular branching structure, the model can identify the branches that are primarily responsible for the observed behavior.

With the latest developments in machine learning and big data, recent works^26,28^ started employing methodologies from these fields to compare the performance of existing decision-making models and create new decision-making models that boast high accuracy in predicting people’s choices. Understandably, these works focus on simple single-stage problems, presumably because this is the domain that most existing models work with and in which data is available in abundance. Multi-stage problems that our model primarily tackles are by comparison put into little consideration by previous modelling and experimental work (For instance, in the experiment by Erev et al.^26^, only 1 question (on St. Petersburg paradox) has sequential properties). One popular problem that we find promising to investigate in the context of our work is the St. Petersburg paradox^76^, as it features a gambling game with explicit stages and payouts that depends on the stage the participant reaches. Existing experimental work that we are aware of only considered the exponentially increasing reward structure proposed in the original form of the paradox, and thus produced a limited dataset. New experimental work that keeps the game structure but varies the reward could provide diversified data to test our model. Techniques from the abovementioned machine-learning-based works^26,28^ can then be applied to quantify the performance of the model, or to produce improved models that consider additional factors in decision-making that our current model does not account for. We hope that our work can catalyze the study of decision-making using problems with more complicated structures, which is an important step in understanding economic decision-making in the highly complex and interactive human society.

### Supplementary Information

Supplementary Information 1.

Supplementary Information 2.

## Data Availability

The study was not pre-registered. No datasets were generated or analyzed during the current study. Figures 1, 5, 6, 7 are generated using MATLAB. Figures 2, 3, 4 are generated using Microsoft Powerpoint. Please refer to the supplementary file for the MATLAB codes used to plot Figs. 1, 5, 6 and 7.
