# Supplementary material for: A multi-stage anticipated surprise model with dynamic expectation for economic decision-making
Source: Sci Rep. 2024 Jan 5;14:657. doi: 10.1038/s41598-023-50529-y (PMC10770108; doi:10.1038/s41598-023-50529-y)
Supplement: Supplementary file 1 — Supplementary Information 1. [file 41598_2023_50529_MOESM1_ESM.pdf]

Online Appendices and Supplementary Information

Appendix 1: Reproducing the patterns of prospect theory in the AS model with surprise functions of general form

Here we are showing that the predictions from the AS model conform to the empirical observation in Kahneman and Tversky (1979) for the type of problems described in Table 1. For the gain domain, it is useful to study how the surprise function varies with respect to p in the setting described in Table A1.

	Reward	Probability
Outcome 1	$1/p$	p
Outcome 2	0	$1-p$

Table A1: The setting for studying the property of the surprise function in the gain domain. Note that we set $\bar{x} = 1$ to simplify analysis.

In this setting, the surprise value Δ, as a function p, is given by:

$$\Delta(p) = p\delta\left(\frac{1}{p} - 1\right) + (1-p)\delta(-1) = pf\left(\frac{1}{p} - 1\right) - (1-p)kf(1) \qquad \text{(A1)}$$

From (A1), we can obtain the following important results:

$$\Delta(p = 1) = 0$$
$$\lim_{p\to 0} \delta(p) = \lim_{p\to 0} pf\left(\frac{1}{p}\right) = \infty$$
$$\frac{\partial \delta}{\partial p}|_{p=1} = kf(1) - f'(0) \geq f(1) - f'(0) > 0$$
$$\frac{\partial^2 \delta}{\partial p^2} = \frac{1}{p^3} f''\left(\frac{1}{p} - 1\right) > 0$$

The 2nd and 4th conditions mean that $\Delta(p)$ is positive and decreasing at small p. The 1st, 3rd and 4th conditions necessitate that $\Delta(p)$ reaches negative value at some intermediate values of p, and then increases and reaches the value of 0 at $p = 1$. This results in a U-shaped $\Delta(p)$ that crosses zero at intermediate p. See, for example, Figure 1.
The value of p where $\Delta(p) = 0$, aside from the trivial solution $p = 1$, corresponds to the probability where the subject switch from the gambling option to the certain option. For the special case where $k = 1$, it can be easily shown from (A1) that $\Delta(p = 0.5) = 0$. For the realistic case where $k > 1$, note that δ is decreasing with k, which means that $\Delta(p = 0.5) < 0$ and that Δ crosses 0 at a smaller value of p.

In the loss domain, the relevant setting is shown in Table A2.

	Reward	Probability
Outcome 1	$-1/p$	p
Outcome 2	0	$1-p$

Table A2: The setting for studying the property of the surprise function in the loss domain.

In this setting, the surprise value Δ_- is given by:

$$\Delta_{-}(p) = pkf\left(\frac{-1}{p} - 1\right) + (1-p)f(-1)$$
$$= -pkf\left(\frac{1}{p} - 1\right) + (1-p)f(1) \qquad \text{(A2)}$$

We are particularly interested in knowing whether reflection effect holds in our model. For a pair options with probability p_1 and p_2 respectively, reflection effect holds when $\Delta(p_1) > \Delta(p_2)$ and $\Delta_{-}(p_1) < \Delta_{-}(p_2)$, or when $\Delta(p_1) < \Delta(p_2)$ and $\Delta_{-}(p_1) > \Delta_{-}(p_2)$. For $k = 1$, $\Delta_{-}(p) = -\Delta(p)$, which implies that the reflection effect can be observed at all values of p, since $\Delta(p_1) > \Delta(p_2)$ implies $\Delta_{-}(p_1) < \Delta_{-}(p_2)$ for all p_1, p_2, and vice versa. For $k > 1$, reflection effect is not always observed. For example, both $\Delta(0.5)$ and $\Delta_{-}(0.5)$ are negative and $\Delta(1) = \Delta_{-}(1) = 0$. So, we have $\Delta(1) > \Delta(0.5)$ and $\Delta_{-}(1) > \Delta_{-}(0.5)$, violating the reflection effect. In general, the reflection effect can be observed for extreme values of p_1 and p_2, while it becomes ambiguous when they take intermediate values.

Appendix 2: Reproducing the results for problem 10 in Kahneman's 1979 paper using the AS model

Here we are showing that our sequential branching mechanism implies that for problem 10 in Kahneman's 1979 paper, consistent to the experiment observations and what PT proposed, it is effectively equivalent to the case when the 1st stage of the problem is ignored. The branching scheme of a general version of problem 10 is shown in Figure S1.

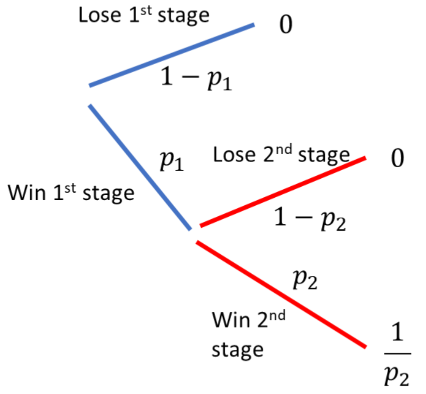

Figure S1: The branching scheme for a general version of problem 10 in Kahneman and Tversky (1979).

The surprise value $\Delta_{two\ stage}$, as a function p_2, is given by:

$$\Delta_{two\ stage}(p_2)$$
$$= (1-p_1)\delta(-p_1) + p_1\delta(1-p_1) + p_1\left(p_2\delta\left(\frac{1}{p_2} - 1\right) + (1-p_2)\delta(-1)\right)$$
$$= f(1-p_1) - (1-p_1)kf(p_1) + p_1\left(p_2 f\left(\frac{1}{p_2} - 1\right) - (1-p_2)kf(1)\right) \qquad \text{(A3)}$$

Note that the term $f(1-p_1) - k(1-p_1)f(p_1)$ is independent of p_2. This is intuitive because events involving the same branch and same expected value at the intermediate states should generate the same anticipated surprise.

Consider two options: one with $p_2 = r$, the other with $p_2 = s$. The difference in the surprise value $D_{two\ stage} = \Delta_{two\ stage}(r) - \Delta_{two\ stage}(s)$ is given by:

$$D_{two\ stage} = p_1\left(rf\left(\frac{1}{r}-1\right) - sf\left(\frac{1}{s}-1\right) - k(r-s)f(1)\right) \qquad \text{(A4)}$$

In Kahneman's 1979 paper, it was suggested that the problem is equivalent to the case when the common blue branch is removed. When the common branch is removed, the problem reverts to the one that we discussed in Section 1. The surprise $\Delta_{one\ stage}(p_2)$ is given by eq. (A1) in Appendix 1. The difference in the surprise value for the same two options $D_{one\ stage} = \Delta_{one\ stage}(r) - \Delta_{one\ stage}(s)$ is given by:

$$D_{one\ stage} = rf\left(\frac{1}{r}-1\right) - sf\left(\frac{1}{s}-1\right) - k(r-s)f(1) \qquad \text{(A5)}$$

The option preference is determined by the sign of D. Here, since $D_{two\ stage}$ only differs from $D_{one\ stage}$ by a multiplicative factor p_1, they always have the same sign. Thus, the model predicts the same preference no matters the choice of r and s, meaning that the two-stage problem and the single-stage problem (obtained by removing the common branch) are practically equivalent in the anticipated surprise model.

Appendix 3: Analysis of Blackjack gambling

The rule of Blackjack

Here we are only describing the rules of the blackjack that are relevant to this work. Also please note that there are no official rules for blackjack as casinos are free to introduce their own house rules. However, the one we describe here is one of the best known, widely used in casinos and work in gambling analysis (Shackleford, 2019a).

Blackjack uses standard 52-card decks. In a casino setting where many games are played, multiple decks of cards are used and played cards are introduced back into the deck often. This is to minimize the change of the winning odds as a result of changes in the composition of the deck as cards are exhausted. The goal of the player is to obtain a hand of cards with a higher value than that of the dealer. The value of the hand depends on a point system. Points are calculated by summing up the numbers on all cards in the hand. Face cards (i.e. Js, Qs and Ks) stands for 10 points. For the Aces, they can stand for either 1 point or 11 points, chosen in order to maximize the value of the hand. The value of a hand in descending order is as follows: an Ace and a single card with 10 points (also known as blackjack (abbrev. BJ)), 21 points (but not BJ), 20 points, 19 points, 18 points, 17 points, 4-16 points, more than 21 points (known as busted hand) for dealer, busted hand for player.

In the beginning of a round, the player is given two cards and the dealer is given one card faced up and one card faced down (the player cannot read the card faced down). The player plays before the dealer, except when the faced-up card of the dealer is an Ace, in which case we will cover later. He can choose to take an extra card or not to. If he chooses to take an extra card, a card will be dealt to him, and the same option will be presented to him again until his hand become busted. If he chooses not to, the turn will be passed to the dealer. The dealer will keep taking extra cards until his hand has more than or equal to 17 points. After the dealer finished playing, the round ends, and whoever has a hand with a higher value wins. When the player wins, he receives twice the amount of his original bet, thus giving him a net win of the size of his bet. An exception is when he wins with a hand of blackjack, in which case he will in addition get an extra amount of 0.5 times of his bet. When the player loses, he receives nothing, thus giving him a net loss of the size of his bet. In the event where the hand

of the player and the dealer has equal value, normally the player will get back his bet and thus winning or losing nothing. However, to simplify our analysis, in section 3.1.1 and 3.1.2.2, we assume that the player will instead throw a coin to decide if he wins or loses, giving him 50% chance of winning and 50% chance of losing an amount equal to the size of his bet.

If the face-up card of the dealer is an Ace, the player will be given an option to place a side bet of the size half his original bet, known as 'the insurance', to bet on whether the hand of the dealer is Blackjack. Before the player starts playing, the dealer will peek at the face-down card. If the dealer has blackjack, the player will get a net win of the amount 2 times of his side bet, which is equivalent to the size of his original bet. At this point, the original bet can also be resolved. If the dealer does not have blackjack, the player will lose his side bets and play resumes in order to resolve the original bet. The placement of the insurance side bet does not affect how the original bet is resolved.

Analysis for Section 3.11 (16 vs 10 situation)

Here we are showing that $\Delta_{stand} \geq \Delta_{hit}$. First, we note that the size of x has no role in affecting the rank of Δ_{bet} and $\Delta_{not\ bet}$ since we can make a transformation to remove x during the comparison between these two quantities. To simplify our analysis, we set $x = 1$ such that
Δ_{stand} and Δ_{hit} is given by:

$$\Delta_{stand} = p_0 f(1-p_0) - (1-p_0) k f(p_0) \quad \text{(A6a)}$$

$$\Delta_{hit} = -\left(1-\frac{p_0}{p_2}\right) k f(p_0) + \frac{p_0}{p_2}\left[f(p_2-p_0) + p_2 f(1-p_2) - (1-p_2) k f(p_2)\right] \quad \text{(A6b)}$$

$$D = \Delta_{stand} - \Delta_{hit} = p_0\left(\frac{1}{p_2}-1\right) k\big(f(p_2)-f(p_0)\big) + p_0\big(f(1-p_0)-f(1-p_2)\big) - \frac{p_0}{p_2} f(p_2-p_0) \quad \text{(A7)}$$

Since f is convex, $\mathrm{f}(p_2-p_0) \leq f(p_2)-f(p_0)$, eq. (A7) can then be rewritten as:

$$D \geq \frac{p_0}{p_2}(k-1)\big(f(p_2)-f(p_0)\big) + p_0\Big[\big(f(1-p_0)+kf(p_0)\big) - \big(f(1-p_2)+kf(p_2)\big)\Big] \quad \text{(A8)}$$

For $k = 1$, the 1st term in eq. (A8) vanishes. For the 2nd term, define $F(z) = f(1-z) + f(z),\ z \in [0,1]$.

Consider the conditions $0 \leq p \leq q \leq \frac{1}{2}$, using the properties of a convex function again, we have

$$f(1-p) - f(1-q) \geq (q-p) f'(1-q)$$

$$f(q) - f(p) \leq (q-p) f'(q) \leq (q-p) f'(1-q) \leq f(1-p) - f(1-q) \quad \text{(A9)}$$

Eq. (A9) means $F(q) > F(p)$ for $0 \leq p \leq q \leq \frac{1}{2}$. For the domain $\frac{1}{2} < z \leq 1$, we can make use of the fact that by definition, $F(z) = F(1-z)$.

In a player's 16 vs dealer's 10 situation, $p_0 = 0.23$, $p_1 = \frac{5}{13}$, $p_2 = \frac{p_0}{p_1} = 0.6$ (Shackleford, 2019b). By above, $f(1-p_0) + f(p_0) = F(0.23) > F(0.4) = F(0.6) = f(1-p_2) + f(p_2)$, and thus $D \geq 0$.

For $k > 1$, we note that $\Delta\delta$ is an increasing function with k since

$$\frac{dD}{dk} = p_0\left(\frac{1}{p_2} - 1\right)\left(f(p_2) - f(p_0)\right) > 0 \quad \text{(A10)}$$

Therefore $D \geq 0$ also holds for $k > 1$.

Analysis for Section 3.1.2.2 (taking side bets when the player has a non-blackjack good hand)

Here we are showing that $\Delta_{bet} \geq \Delta_{no\ bet}$. We are assuming that both the options of taking and not taking side bets has the same expected value, this amounts to $p_1 = \frac{1}{3}$ (in reality p_1 is slightly different at $p_1 = \frac{4}{13}$). Denoting the initial expected value of the hand, i.e before the dealer peeks for BJ by E_0. E_0 can be expressed as

$$E_0 = (1 - p_1)\left[\frac{x}{2}p_2 - \frac{3x}{2}(1 - p_2)\right] = \frac{2x}{3}\left(2p_2 - \frac{3}{2}\right) = x\left(\frac{4}{3}p_2 - 1\right) \quad \text{(A11a)}$$

Like in the previous section, the size of x has no role in affecting the rank of Δ_{bet} and $\Delta_{not\ bet}$ since we can make a transformation to remove x during the comparison between these two quantities. To simplify our analysis, we set $x = 1$ such that

$$E_0 = \frac{4}{3}p_2 - 1 \quad \text{(A11b)}$$

Let E_1 be the expected value after the dealer peeks for BJ if the player takes the side bet.

$$E_1 = \frac{E_0}{(1-p_1)} = \frac{3}{2}E_0 \quad \text{(A12)}$$

Let E_2 be the expected value after the dealer peeks for BJ if the player does not take the side bet.

$$E_2 = E_1 + \frac{1}{2} = \frac{3}{2}E_0 + \frac{1}{2} \quad \text{(A13)}$$

It is trivial that $E_2 \geq E_0$. However, whether it is $E_1 \geq E_0$ or $E_1 \leq E_0$ depends on the sign of E_0. Therefore, we divide the problem in the separate cases: $E_0 < 0$ and $E_0 \geq 0$.

Case 1: $E_0 < 0$

When $E_0 < 0$, $E_1 < E_0$. Δ_{bet} and $\Delta_{no\ bet}$ can be expressed as

$$\Delta_{bet} = p_1 g f(-E_0) + (1 - p_1)\left[-kf\left(\frac{-E_0}{2}\right) + p_2 f\left(\frac{1}{2} - \frac{3E_0}{2}\right) - (1 - p_2)kf\left(\frac{3}{2} + \frac{3E_0}{2}\right)\right] \quad \text{(A14a)}$$

$$\Delta_{no\ bet} = -p_1 k f(1 + E_0) + (1 - p_1)\left[f\left(\frac{1}{2} + \frac{E_0}{2}\right) + p_2 f\left(\frac{1}{2} - \frac{3E_0}{2}\right) - (1 - p_2)k_1 f\left(\frac{3}{2} + \frac{3E_0}{2}\right)\right] \quad \text{(A14b)}$$

Substituting in $p_1 = \frac{1}{3}$, we have

$$D = \Delta_{bet} - \Delta_{no\ bet} = \frac{1}{3}\left[\left(f(-E_0) + kf(1 + E_0)\right) - 2\left(kf\left(\frac{-E_0}{2}\right) + f\left(\frac{1}{2}(1 + E_0)\right)\right)\right] \quad \text{(A15)}$$

For $k = 1$, using the properties of a convex function, we have

$f(-E_0) \geq 2f\left(\frac{-E_0}{2}\right)$ and $f(1 + E_0) \geq 2f\left(\frac{1}{2}(1 + E_0)\right)$.

It follows that $D \geq 0$.

For $k > 1$, we again study the derivative of $\Delta\delta$ with respective to k

$$\frac{dD}{dk} = \frac{1}{3}\left[f(1+E_0) - 2f\left(\frac{-E_0}{2}\right)\right] \quad \text{(A16)}$$

If $E_0 \geq \frac{-1}{2}$, we have $f(1+E_0) \geq 2f\left(\frac{1}{2}(1+E_0)\right) \geq 2f\left(\frac{-E_0}{2}\right)$ such that D increases with k and remains positive at $k > 1$.

From eq. (A11b), $E_0 \geq \frac{-1}{2}$ when $p_2 \geq \frac{3}{8}$. Since a large p_2 means that there is a large probability of winning with the hand, which, in order words, means the hand is a good hand. The results suggest that players prefer to take side bets when they have good hands.

Case 2: $E_0 \geq 0$

Since in the previous section, we established that $E_0 \geq \frac{-1}{2}$ is always considered good hands, we have to show that when $E_0 > 0$, $\Delta\delta \geq 0$ unconditionally. Noting that when $E_0 \geq 0$, $E_1 \geq E_0$, and that from eq. (A11b) E_0 has an upper bound of $\frac{1}{3}$, Δ_{bet} and $\Delta_{no\ bet}$ is given by

$$\Delta_{bet} = -p_1 k f(E_0) + (1-p_1)\left[f\left(\frac{E_0}{2}\right) + p_2 f\left(\frac{1}{2} - \frac{3E_0}{2}\right) - (1-p_2)kf\left(\frac{3}{2} + \frac{3E_0}{2}\right)\right] \quad \text{(A17a)}$$

$$\Delta_{no\ bet} = -p_1 k f(1+E_0) + (1-p_1)\left[f\left(\frac{1}{2} + \frac{E_0}{2}\right) + p_2 f\left(\frac{1}{2} - \frac{3E_0}{2}\right) - (1-p_2)kf\left(\frac{3}{2} + \frac{3E_0}{2}\right)\right] \quad \text{(A17b)}$$

Substituting in $p_1 = \frac{1}{3}$, we have

$$D = \Delta_{bet} - \Delta_{no\ bet} = \frac{1}{3}\left[k\big(f(1+E_0) - f(E_0)\big) - 2\left(f\left(\frac{1}{2} + \frac{E_0}{2}\right) - f\left(\frac{E_0}{2}\right)\right)\right] \quad \text{(A18)}$$

For $k = 1$, again using the properties of a convex function, we have

$$f(1+E_0) - f\left(\frac{1}{2} + \frac{E_0}{2}\right) > \left(\frac{1}{2} + \frac{E_0}{2}\right) f'\left(\frac{1}{2} + \frac{E_0}{2}\right)$$
$$f\left(\frac{1}{2} + \frac{E_0}{2}\right) - f\left(\frac{E_0}{2}\right) < \frac{1}{2} f'\left(\frac{1}{2} + \frac{E_0}{2}\right)$$
$$f(E_0) - f\left(\frac{E_0}{2}\right) < \left(\frac{E_0}{2}\right) f'(E_0) < \frac{E_0}{2} f'\left(1 + \frac{E_0}{2}\right)$$

This gives

$$f(1+E_0) - f\left(\frac{1}{2} + \frac{E_0}{2}\right) > f\left(\frac{1}{2} + \frac{E_0}{2}\right) - f\left(\frac{E_0}{2}\right) + f(E_0) - f\left(\frac{E_0}{2}\right)$$
$$f(1+E_0) - f(E_0) > 2\left(f\left(\frac{1}{2} + \frac{E_0}{2}\right) - 2f\left(\frac{E_0}{2}\right)\right)$$

It follows that $D \geq 0$.

From (A18), it is obvious that D increases with k such that $D \geq 0$ still holds when $k > 1$.

Appendix 4: Analysis of the Ellsberg paradox

Here we are exploring the condition in which the unambiguous urn would be preferred over the ambiguous urn. We assume that a symmetric prior probability that satisfies $p(m) = p(1-m)$ for $m \in \left\{0, \frac{1}{2n}, \frac{2}{2n}, \ldots, 1\right\}$. The surprise value for picking the unambiguous urn (Δ_1) and the ambiguous urn (Δ_2) is given by eqs. (6a) and (6b) in the main text. For convenience, we are repeating them here:

$\Delta_1 = \frac{1}{2}\delta\left(\frac{1}{2}\right) + \frac{1}{2}\delta\left(-\frac{1}{2}\right) = \frac{1}{2}f\left(\frac{1}{2}\right) - \frac{k}{2}f\left(\frac{1}{2}\right)$
$= \sum_m p(m)\left[\frac{1-k}{2}f\left(\frac{1}{2}\right)\right]$
$= (1-k)\sum_{m<1/2} p(m)f\left(\frac{1}{2}\right) + p\left(\frac{1}{2}\right)\frac{1-k}{2}f\left(\frac{1}{2}\right)$ (A19a)
$\Delta_2 = \sum_m p(m)\left[\delta\left(m-\frac{1}{2}\right) + m\delta(1-m) + (1-m)\delta(-m)\right]$
$= \sum_{m<1/2} p(m)\left[-kf\left(\frac{1}{2}-m\right) + mf(1-m) - k(1-m)f(m)\right] + p\left(\frac{1}{2}\right)\frac{1-k}{2}f\left(\frac{1}{2}\right) +$
$\sum_{m>1/2} p(m)\left[f\left(m-\frac{1}{2}\right) + mf(1-m) - k(1-m)f(m)\right]$
$= \sum_{m<1/2} p(m)\left[-kf\left(\frac{1}{2}-m\right) + mf(1-m) - k(1-m)f(m)\right] + p\left(\frac{1}{2}\right)\frac{1-k}{2}f\left(\frac{1}{2}\right)$
$+ \sum_{m<1/2} p(1-m)\left[f\left(\frac{1}{2}-m\right) + (1-m)f(m) - kmf(1-m)\right]$
$= (1-k)\sum_{m<1/2} p(m)\left[f\left(\frac{1}{2}-m\right) + mf(1-m) - (1-m)f(m)\right] + \left(\frac{1}{2}\right)\frac{1-k}{2}f\left(\frac{1}{2}\right)$ (A19b)

Note that we used $\sum_{m>1/2} F(1-m) = \sum_{m<1/2} F(m)$ for general function F.

$D = \Delta_2 - \Delta_1$
$= (1-k)\sum_{m<1/2} p(m)\left[f\left(\frac{1}{2}-m\right) + mf(1-m) - (1-m)f(m) - f\left(\frac{1}{2}\right)\right]$ (A20)

Condition 1: f is not strongly convex

Since $k \geq 1$, a sufficient condition for $D > 0$ is

$f\left(\frac{1}{2}-m\right) + mf(1-m) + (1-m)f(m) > f\left(\frac{1}{2}\right)\ \forall 0 \leq m \leq \frac{1}{2}$ (A21)

Define $F(m) = f\left(\frac{1}{2}-m\right) + mf(1-m) + (1-m)f(m)$. Note that

$F(0) = F\left(\frac{1}{2}\right) = f\left(\frac{1}{2}\right)$
$F'(m) = -f'\left(\frac{1}{2}-m\right) - mf'(1-m) + f(1-m) + (1-m)f'(m) - f(m)$
$F'\left(\frac{1}{2}\right) = -f'(0) < 0$

Based on these results, since f (and hence F) is a continuous function, (A21) would be true if $F''(m) < 0\ \forall 0 \leq m \leq \frac{1}{2}$.

$F''(m) = (1-m)f''(m) + mf''(1-m) + f''\left(\frac{1}{2}-m\right) - 2f'(1-m) - 2f'(m)$ (A22)

It is not true that $F''(m) < 0$ at $0 \leq m \leq \frac{1}{2}$ for any convex function f. Here we would explore possible conditions where $F''(x) < 0$ is true. We note that

$F''(m)$
$= (1-m)f''(m) + mf''(1-m) + f''\left(\frac{1}{2}-m\right) - 2f'(1-m) - 2f'(m)$
$= (1-m)[f''(m) - 2f'(m)] + m[f''(1-m) - 2f'(1-m)] - \left[2mf'(m) +\right.$
$\left.(2-2m)f'(1-m) - f''\left(\frac{1}{2}-m\right)\right]$ (A23)

Note that if $f''(m) \leq \frac{3}{2}f'(m)$, when $0 \leq m < \frac{1}{4}$,

$2mf'(m) + (2-2m)f'(1-m) > \frac{3}{2}f'(1-m) \geq \frac{3}{2}f'\left(\frac{1}{2}-m\right) \geq f''\left(\frac{1}{2}-m\right)$,

and when $\frac{1}{4} \leq m \leq \frac{1}{2}$,

$$2mf'(m) + (2-2m)f'(1-m) \geq 2f'(m) \geq 2f'\left(\frac{1}{2}-m\right) > f''\left(\frac{1}{2}-m\right).$$

Therefore, one can infer from eq. (A23) that

$$F''(m) < 0 \text{ if } f''(m) \leq \frac{3}{2}f'(m)\ \forall 0 \leq m \leq \frac{1}{2}. \quad \text{(A24)}$$

Please note that eq. (A24) is a sufficient condition but not a necessary condition for $D > 0$. As shown in Figure 5, there are functions f which do not fulfil eq. (A24) but still allows $D > 0$.
Eq. (A24) suggests that if is not very convex then, $D > 0$ is guaranteed. On the other hand, $D > 0$ can also be achieved if f is strongly convex, as we will show below.

Condition 2: f is strongly convex

We investigate what happens if f is strongly convex. Starting from eq. (A20),

$$\begin{aligned} D &= (k-1)\textstyle\sum_{m<1/2} p(m)\left[f\left(\frac{1}{2}-m\right) + mf(1-m) + (1-m)f(m) - f\left(\frac{1}{2}\right)\right] \\ &> (k-1)\textstyle\sum_{m<1/2} p(m)\left[mf(1-m) - f\left(\frac{1}{2}\right)\right] \end{aligned} \quad \text{(A25)}$$

because $mf(1-m)$ would dominate the other neglected terms for strongly convex f.

Next, we make use of the fact that f is convex. There are multiple ways of doing it, which will lead to different constraints on f for ambiguity aversion. Here, we show an example.

Applying the Jensen's inequality, we have $\mathrm{E}^{(1)}[f(1-m)] \geq f(1-\mathrm{E}^{(1)}[m])$,
where $\mathrm{E}^{(c)}[\cdot] \equiv \frac{\sum_{m<1/2}\ \cdot\ m^c\, p(m)}{\sum_{m<1/2} m^c\, p(m)}$. This leads to

$$D > (k-1)\left(\textstyle\sum_{m<1/2} p(m)\right)\left[\mathrm{E}^{(0)}[m] f\left(1-\mathrm{E}^{(1)}[m]\right) - f\left(\frac{1}{2}\right)\right]. \quad \text{(A26)}$$

Note that $0 < \mathrm{E}^{(c)}[m] < \frac{1}{2}$ since $0 < m < 1/2$ for both $c = 0$ and 1. Hence, $D > 0$ is guaranteed if f is sufficiently convex. More specifically,

$$\mathrm{E}^{(0)}[m] f\left(1-\mathrm{E}^{(1)}[m]\right) > f\left(\frac{1}{2}\right) \quad \text{(A27)}$$

For example, for $f(m) = m^r$ and $p(m) = \frac{1}{2n+1}$ for all m, one can easily show that $D > 0$ if

$$r > \frac{\log\frac{4n}{n-1}}{\log\left(\frac{4n+1}{3n}\right)}, \quad \text{(A28)}$$

which implies a strongly convex function f with $r > 4.82$ in the limit of large n. We would like to emphasize again that eqs. (A27) and (A28) are not necessary condition for $D > 0$. In fact, as we have shown in our numerical study (Figure 5), $D > 0$ can be achieved with a much less strongly convex function than eqs. (A27) and (A28) suggest.

Appendix 5: Analysis on CPT and RT on examples in Section 3

Blackjack side-bet taking for good hands (Section 3.1.2.2)

The options and their outcomes, arranged in rank order, in reorganized in Table A3.

Reward for Option 1 (Side bets)	Reward for Option 2 (No side bets)	Probability
$-3/2$	-1	$2/3-p$
0	0	$1/3$
$1/2$	1	p

Table A3: The possible outcomes and their probability for taking and not taking side bets. p is the probability of winning the hand, where $p=\frac{2}{3}p_2$ in the main text.

RT

For RT, anticipated regret R is given by $R=\sum_i p_i\, Q(u(y_i)-u(x_i))$, where x_i is the outcome for the chosen option, y_i is the outcome if the other option is chosen, p_i is the probability of the outcomes, u is a concave, odd function, Q is a convex, odd function (Bleichrodt & Wakker, 2015). The option that minimizes R is chosen. Because of the symmetry in Q, only terms where $x_i>y_i$ need to be considered.

The regret for taking the side bets is given by

$$R_{side\ bets}=\frac{2}{3}p_2Q\left(u(1)-u(\tfrac{1}{2})\right)+\frac{2}{3}(1-p_2)Q\left(u\left(\frac{3}{2}\right)-u(1)\right) \quad \text{(A29)}$$

Again, we have set $x=1$ as in Appendix 3.

Since u is concave, $u(1)>\frac{1}{2}\left(u\left(\frac{1}{2}\right)+u\left(\frac{3}{2}\right)\right)$, and hence $u(1)-u\left(\frac{1}{2}\right)>u\left(\frac{3}{2}\right)-u(1)$ such that $R_{side\ bets}$ is increasing with p_2. This means that RT predicts that players have less tendency to take side bets when they have good hands than when they have bad hands, which is at odds with experimental observations.

CPT

The prospect V for taking and not taking side bets is given by

$$V_{side\ bets}=w\left(\frac{2}{3}-p\right)v\left(-\frac{3}{2}\right)+\left(w(1-p)-w\left(\frac{2}{3}-p\right)\right)v(0)+\left(w(1)-w(1-p)\right)v\left(\frac{1}{2}\right) \quad \text{(A30a)}$$

$$V_{no\ side\ bets}=w(1-p)v(-1)++\left(w(1)-w(1-p)\right)v(1), \quad \text{(A30b)}$$

where $v(x)=x^r$ with $r<1$, $v(-x)=-kv(x)$ with $k>0$, for all $x\geq 0$, and $w(p)=\frac{p^\gamma}{(p^\gamma+(1-p)^\gamma)^{\frac{1}{\gamma}}}$ with $\gamma<1$. Please note that we have made the simplifying assumption that the the concavity of v and the value of γ is the same for the gain and loss domain. This simplification would not lead to qualitative difference in the analysis (results not shown). For details, please refer to Tversky and Kahneman (1992).

The difference in the prospect between the options D_{CPT} is given by

$$D_{CPT}=V_{side\ bets}-V_{no\ side\ bets}=k\left(w(1-p)v(1)-w\left(\frac{2}{3}-p\right)v\left(-\frac{3}{2}\right)\right)-\left(w(1)-w(1-p)\right)\left(v(1)-v\left(\frac{1}{2}\right)\right) \quad \text{(A31)}$$

We have plotted D_{CPT} against p for a variety of parameters γ, k and r, as shown in Figure S2.

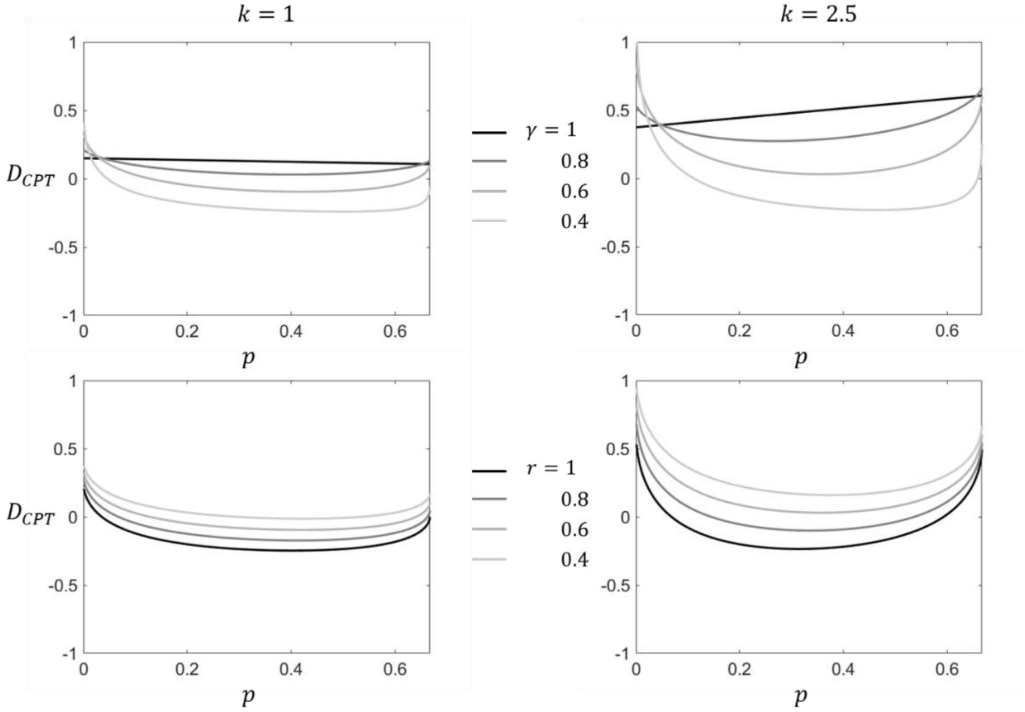

Figure S2: Differences in prospect between taking and not taking side bets (D_{CPT}) at different p for different choices of parameters. Positive D_{CPT} implies preference for taking side bets while large p implies good hands. Top: r is fixed at 0.6 while the lines in the plots correspond to different values of γ. Bottom: Top: γ is fixed at 0.6 while the lines in the plots correspond to different values of r . Left: $k = 1$. Right: $k = 2.5$. Note that no combination of parameters is consistent with the experimental observation that taking side bets is preferred when the player has a good hand.

It can be observed that both the concavity of the value function r and probability weighting γ raised D at small p. The latter also suppressed D at large p. Overall, this is again at odds with experimental results where D should be increasing with p and should not be positive at all p. As a comparison, the differences in anticipated surprise predicted by our model D (See eq. (A15)) using realistic parameters we suggested at Section 3.3 in the main text are shown in Figure S3. Consistent with our analysis at Appendix 3 and the experimental observations, D is increasing with p and is positive at large p.

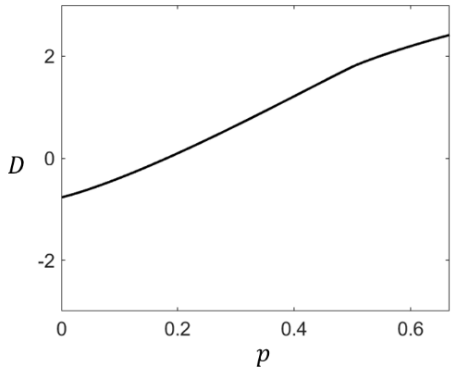

Figure S3: Differences in anticipated surprise based on the model in this work between taking and not taking side bets (D) at different p. Positive D implies preference for taking side bets.

Our model prediction is consistent with the experimental observation that taking side bets is preferred when the player has a good hand. We set $f(x) = x^r$, where $r = 1.5$, and $k = 2.5$.

Allais paradox (Section 3.3)

For convenience, we state the problems in the original version of Allais paradox (i.e. Table 2) here again in Table A4.

(a) Problem 1

	Option 1		Option 2 *	
	Reward	Probability	Reward	Probability
Outcome 1	0	0.89	0	0.9
Outcome 2	1	0.11	5	0.1

(b) Problem 2

	Option 1 *		Option 2	
	Reward	Probability	Reward	Probability
Outcome 1	1	1	0	0.01
Outcome 2			1	0.89
Outcome 3			5	0.1

Table A4: The two decision-making problems in the Allais paradox. The asterisk depicts the option preferred by most people.

RT

For Problem 1, the regret for the options is given by:

$$R_{Option\,1} = 0.089Q\big(u(5)\big) + 0.011Q(u(5) - u(1)) \quad \text{(A32a)}$$
$$R_{Option\,2} = 0.099Q(u(1)) \quad \text{(A32b)}$$

Option 2 is chosen implies that

$$0.089Q\big(u(5)\big) + 0.011Q(u(5) - u(1)) > 0.099Q(u(1)) \quad \text{(A33)}$$

For Problem 2, the regret for the options is given by:

$$R_{Option\,1} = 0.1Q(u(5) - u(1)) \quad \text{(A34a)}$$
$$R_{Option\,2} = 0.01Q(u(1)) \quad \text{(A34b)}$$

Option 1 is chosen implies that $Q(u(1)) > 10Q(u(5) - u(1))$ (A35)

If $u(5) < 2u(1)$, $u(5) - u(1) < u(1)$. eq. (A35) can be fulfilled by choosing a sufficiently convex function Q. To fulfil eq. (A33), a sufficient condition is $\frac{Q(u(5))}{Q(u(1))} > \frac{0.099}{0.089}$, which also requires Q to be sufficiently convex.

These results show that RT is not theoretically inconsistent with Allais Paradox.

CPT

For Problem 1, the prospect for the options is given by:

$$V_{Option\,1} = w(0.9)v(0) + \big(w(1) - w(0.9)\big)v(5) \quad \text{(A36a)}$$

$$V_{Option\ 2} = w(0.89)v(0) + \big(w(1) - w(0.89)\big)v(1) \qquad \text{(A36b)}$$

Option 2 is chosen implies that $v(5) > \frac{1-w(0.89)}{1-w(0.9)} v(1)$ (A37)

For Problem 2, the regret for the options is given by:

$$R_{Option\ 1} = w(0.01)v(0) + \big(w(0.9) - w(0.01)\big)v(1) + \big(1 - w(0.9)\big)v(5) \qquad \text{(A38a)}$$

$$R_{Option\ 2} = v(1) \qquad \text{(A38b)}$$

Option 1 is chosen implies that $v(5) < \frac{1-w(0.9)+w(0.01)}{1-w(0.9)} v(1)$ (A39)

One can easily see that eqs. (A37) and (A39) can be simultaneously fulfilled by imposing the condition $\frac{1-w(0.89)}{1-w(0.9)} < \frac{v(5)}{v(1)} < \frac{1-w(0.9)+w(0.01)}{1-w(0.9)}$, which, despite posing strict restriction on the parameters in v and w, is not theoretically inconsistent.

Appendix 6: The effect of grouping on the surprise value for the Allais paradox and Birnbaum problem

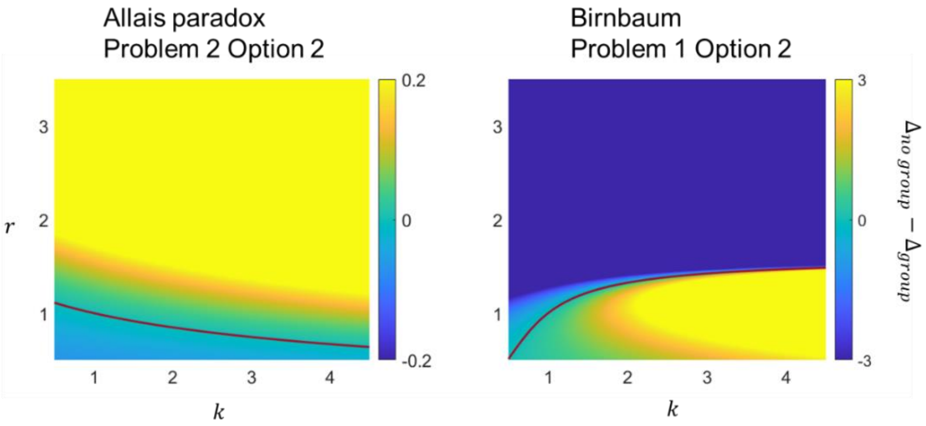

Figure S4: The difference in surprise values between the branching scheme when event grouping is present and when it is absence for Option 2 in the Allais paradox Problem 2 (left) and Option 2 in Problem 1 adopted from Birnbaum (2008) (right), which are discussed in the main text. The dark red line is the boundary where the options are equally preferred, i.e. $\Delta_{group} = \Delta_{no\ group}$. Consistent with our analysis, event grouping reduces the appeal for Option 2 in the Allais paradox for all $k, r > 1$. On the other hand, event grouping increases the appeal for Option 2 in the Birnbaum problem with a slightly more stringent condition that r is not too close to 1 and k is not too large. As with the rest of our numerical studies on these problems, we set $f(x) = x^r$.

Reference (Appendices)

Bleichrodt, H., & Wakker, P. P. (2015). Regret theory: A bold alternative to the alternatives. Economic Journal, 125(583), 493–532.

Kahneman, D., & Tversky, A. (1979). Prospect Theory: An Analysis of Decision under Risk. *Econometrica*, *47*(2), 263–292.

Shackleford, M. (2019a). Blackjack. https://wizardofodds.com/games/blackjack/basics/#rules

Shackleford, M. (2019b). Blackjack Hand Calculator. https://wizardofodds.com/games/blackjack/hand-calculator/

Tversky, A., & Kahneman, D. (1992). Advances in Prospect Theory: Cumulative Representation of Uncertainty. *Journal of Risk and Uncertainty*, *5*(4), 297–323.
