# Supplementary material for: A multi-stage anticipated surprise model with dynamic expectation for economic decision-making
Source: Sci Rep. 2024 Jan 5;14:657. doi: 10.1038/s41598-023-50529-y (PMC10770108; doi:10.1038/s41598-023-50529-y)
Supplement: Supplementary file 2 — Supplementary Information 2. [file 41598_2023_50529_MOESM2_ESM.pdf]

MATLAB Code for Figure 1

```
clear all;
k=2.5;
for i=1:100
    x(i)=0.01*i;
    delta1(i)=x(i)*g(1/x(i)-1)-(1-x(i))*g(1);
    delta2(i)=x(i)*g(1/x(i)-1)-(1-x(i))*k*g(1);
    delta3(i)=-x(i)*g(1/x(i)-1)+(1-x(i))*g(1);
    delta4(i)=-k*x(i)*g(1/x(i)-1)+(1-x(i))*g(1);
    delta5(i)=0;
end

plot(x,delta1,'-r',x,delta2,'--r',x,delta3,'-b',x,delta4,'--
b',x,delta5,'-k');
axis([0 1 -2 2]);
function y=g(x)
    y=x^1.5;
end
```

MATLAB Code for Figure 5

```
clear all;
r(1)=0.5;
for k=1:100
    m=0;
    x(k)=0;
    if (k>1)
        r(k)=r(k-1)+0.1;
    end
    for i=0:50
        m=m+0.01;
        x(k)=x(k)+(0.5-m)^r(k)+m*((1-m)^r(k))+(1-m)*(m^r(k));
        x(k)=real(x(k));
    end
    x(k)=1.5*(x(k)/101-51/101*(0.5^r(k)));
end
plot(r,x);
ylim([-0.02 0.1]);
```

MATLAB Code for Figure 6

```
clear all;
E1=0.11;
E2=0.5;
E3=1;
```

```
E4=1.39;
E5=5/1.1;
x1=1;
x2=0;
x3=5;
a=0.5;
for i=1:1000
    k=0.5;
    for j=1:1000
        %problem 1 option 1
        d1(i,j)= 0.11*delta(x1,E1,k,a)+0.89*delta(x2,E1,k,a);
     %problem 1 option 2
     d2(i,j)= 0.1*delta(x3,E2,k,a)+0.9*delta(x2,E2,k,a);
        %problem 2 option 1
        d3(i,j)=0;
     %problem 2 option 2 without grouping
     d4(i,j)=0.89*delta(x1,E4,k,a)+0.01*delta(x2,E4,k,a)+0.1*delta(x3,
     E4,k,a);
     %problem 2 option 2 with grouping
     d5(i,j)=0.89*delta(x1,E4,k,a)+0.11*delta(E5,E4,k,a)+0.1*delta(x3,
     E5,k,a)+0.01*delta(x2,E5,k,a);
   %     diff(i,j)=d1(i,j)-d2(i,j);
   %     diff(i,j)=d3(i,j)-d4(i,j);
   %     diff(i,j)=d3(i,j)-d5(i,j);
        diff(i,j)=d4(i,j)-d5(i,j);
        if (i==1)
            x(j)=k;
        end
        k=k+0.005;
    end
    y(i)=a;
    a=a+0.004;
end

s=pcolor(x,y,diff)
xlim([0.5 4.5]);
ylim([0.5 3.5]);
colormap parula;
caxis([-0.2 0.2]);
colorbar
s.EdgeColor = 'none';

hold on

t=contour(x,y,diff,[0 0])
xlim([0.5 4.5]);
ylim([0.5 3.5]);
caxis([-0.2 0.2]);

hold off

alpha(0.5)
```

```
function y=delta(x1,x2,k,a)
    if ((x1-x2)>=0)
        y=real((x1-x2)^a);
    else
        y=real(-k*((x2-x1)^a));
    end
end
```

MATLAB Code for Figure 7

```
clear all;
E1=92.5;
E2=95.35;
E3=69;
x1=100;
x2=50;
x3=7;
a=0.5;
for i=1:1000
    k=0.5;
    for j=1:1000
   %Option 1
     d1(i,j)= 0.85*delta(x1,E1,k,a)+0.15*delta(x2,E1,k,a);
   %Option 2 without grouping
    d2(i,j)= 0.95*delta(x1,E2,k,a)+0.05*delta(x3,E2,k,a);
     %Option 2 with grouping
     d3(i,j)=0.85*delta(x1,E2,k,a)+0.15*delta(E3,E2,k,a)+0.1*delta(x1,
     E3,k,a)+0.05*delta(x3,E3,k,a);
       % diff(i,j)=d1(i,j)-d2(i,j);
       % diff(i,j)=d1(i,j)-d3(i,j);
       diff(i,j)=d2(i,j)-d3(i,j);
        if (i==1)
            x(j)=k;
        end
        k=k+0.005;
    end
    y(i)=a;
    a=a+0.004;
end
s=pcolor(x,y,diff)
xlim([0.5 4.5]);
ylim([0.5 3.5]);
caxis([-3 3]);
%caxis([2.85 2.85001])
%caxis([-0.00001 0.00001])
colorbar
s.EdgeColor = 'none';

hold on
```

```
t=contour(x,y,diff,[0 0])
xlim([0.5 4.5]);
ylim([0.5 3.5]);
caxis([-3 3]);
hold off

alpha(0.5)

function y=delta(x1,x2,k,a)
    if ((x1-x2)>=0)
        y=real((x1-x2)^a);
    else
        y=real(-k*((x2-x1)^a));
    end
end
```
